# Supplementary figures and images for: Limitations of multiexponential T1 mapping of cortical myeloarchitecture
Source: PLoS One. 2025 Dec 4;20(12):e0338035. doi: 10.1371/journal.pone.0338035 (PMC12677506; doi:10.1371/journal.pone.0338035)

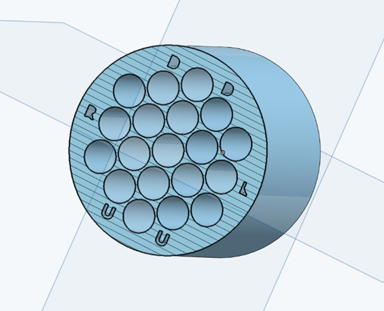

Supplement: S1 Fig — (TIF) [file pone.0338035.s001.tif]
