# Supplementary material for: Limitations of multiexponential T1 mapping of cortical myeloarchitecture
Source: PLoS One. 2025 Dec 4;20(12):e0338035. doi: 10.1371/journal.pone.0338035 (PMC12677506; doi:10.1371/journal.pone.0338035)
Supplement: S2 File — (DOCX) [file pone.0338035.s002.docx]

## Alignment Algorithm Description

Each optimizer returns an estimate of several coefficient pairs (*T*_1_ and *A*_0_). The model used is the same for all the methods: a sum of exponential functions, each with time constants *T*_1_ and relative weight *A*_0_. The order of these exponential functions returned by the model does not necessarily have to correspond with the ground-truth order. Therefore, it is necessary to match the fitted coefficients with the ground-truth ones. This is done using the following algorithm:

1. Create a set of all possible permutations of the estimated coefficients.
2. Compute the distance between all permutations and ground-truth coefficients.
3. Assign the fitted coefficient to the ground-truth coefficient with the lowest distance.

The chosen distance metric is the sum of relative errors of individual components in a given permutation.
