# Supplementary material for: Limitations of multiexponential T1 mapping of cortical myeloarchitecture
Source: PLoS One. 2025 Dec 4;20(12):e0338035. doi: 10.1371/journal.pone.0338035 (PMC12677506; doi:10.1371/journal.pone.0338035)
Supplement: S3 File — (DOCX) [file pone.0338035.s003.docx]

## Modified Maximum Mean Error

Assuming a ground-truth value of *y* estimated by an estimate *x*, the modified maximum mean error *f*(*x*, *y*) can be computed as:

|  | $f\left( x, y \right)=\left\{ \begin{aligned} 0, &x=y=0 \\ \frac{\left\vert y-x \right\vert}{max\left( x, y \right)}, &otherwise \end{aligned} \right.$ | (1) |
| --- | --- | --- |
