# Supplementary material for: Limitations of multiexponential T1 mapping of cortical myeloarchitecture
Source: PLoS One. 2025 Dec 4;20(12):e0338035. doi: 10.1371/journal.pone.0338035 (PMC12677506; doi:10.1371/journal.pone.0338035)
Supplement: S4 File — (PDF) [file pone.0338035.s004.pdf]

Full results of regression analysis

**Method**	**ILT**	**MUL**	**TOM**
**Intercept**	82.499	45.772	33.347
**Intercept standard error**	4.224	2.484	2.388
**Intercept confidence interval (2.5 % - 97.5 %)**	74.197 – 90.801	40.890 – 50.654	28.654 – 38.401
**Intercept p-value**	<0.001	<0.001	<0.001
**Mean ground truth T1 ratio**	-16.846	-9.721	-4.153
**Mean ground truth T1 ratio standard error**	1.659	0.975	0.938
**Mean ground truth T1 ratio confidence interval (2.5 % - 97.5 %)**	-20.106 – -13.586	-11.638 – -7.804	-5.996 – -2.310
**Mean ground truth T1 ratio p-value**	<0.001	<0.001	<0.001
**Number of observations**	432	432	432
**R2**	0.193	0.188	0.044
**R2 Adj.**	0.192	0.186	0.041
**AIC**	4241.1	3782.5	3748.3
**BIC**	4253.3	3794.6	3760.5
**Log. Lik.**	-2117.534	-1888.195	-1871.149
**RMSE**	32.55	19.14	18.40
