# Supplementary material for: Limitations of multiexponential T1 mapping of cortical myeloarchitecture
Source: PLoS One. 2025 Dec 4;20(12):e0338035. doi: 10.1371/journal.pone.0338035 (PMC12677506; doi:10.1371/journal.pone.0338035)
Supplement: S5 File — (PDF) [file pone.0338035.s005.pdf]

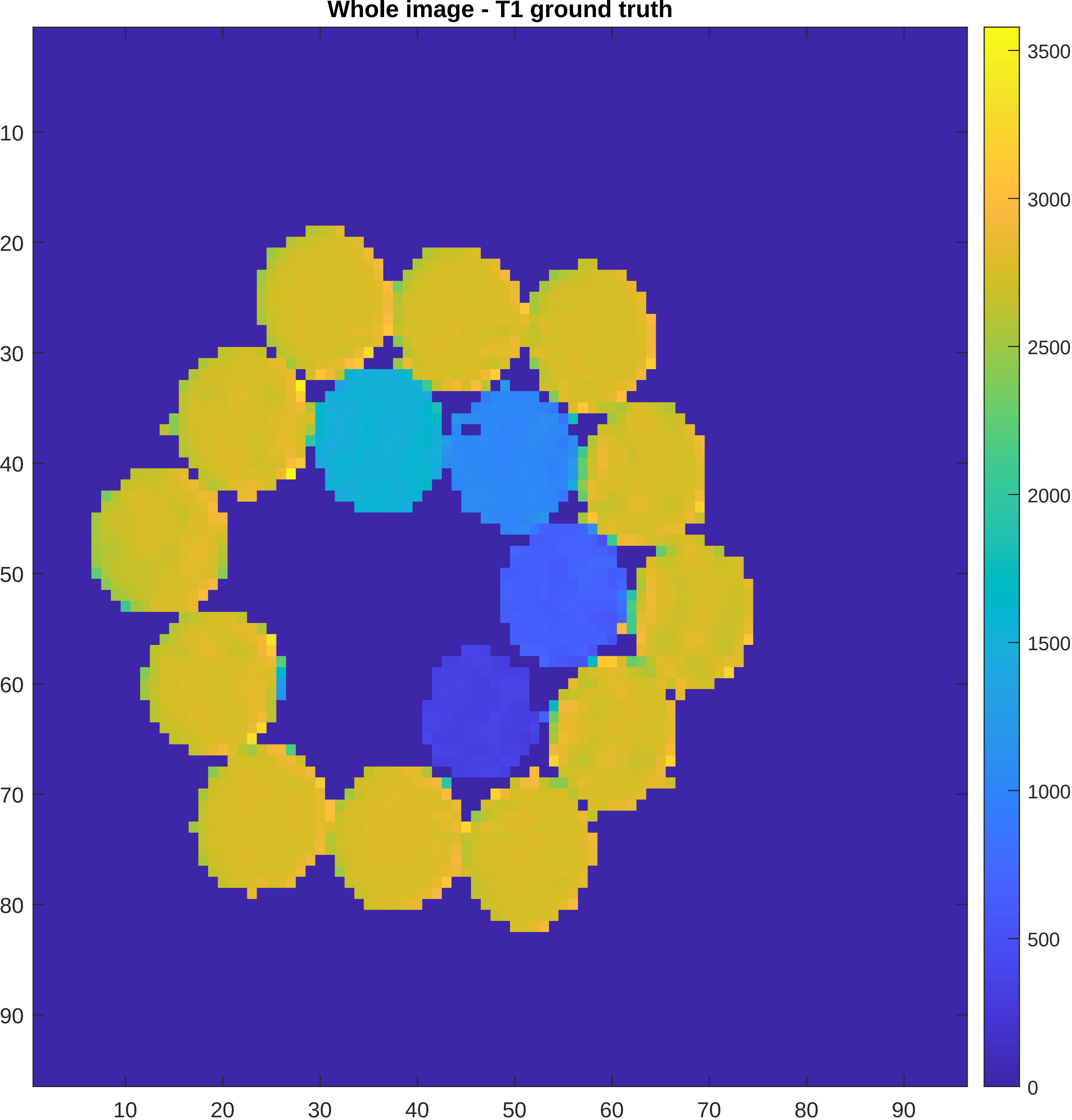
Whole image - T1 ground truth
10
20
30
40
50
60
70
80
90
0
500
1000
1500
2000
2500
3000
3500

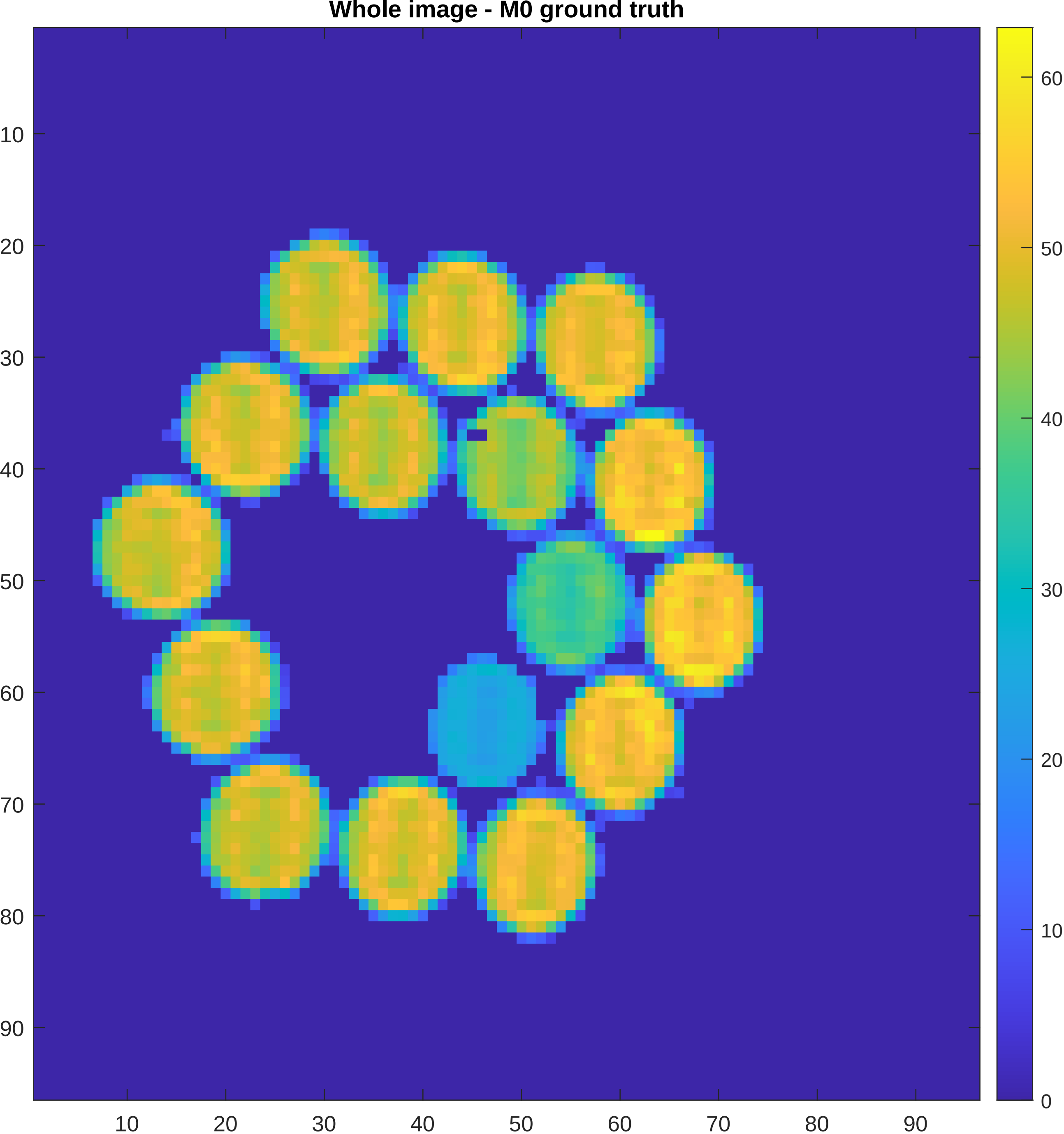
Whole image - M0 ground truth
10
20
30
40
50
60
70
80
90
0
10
20
30
40
50
60

Comb-001,ILT

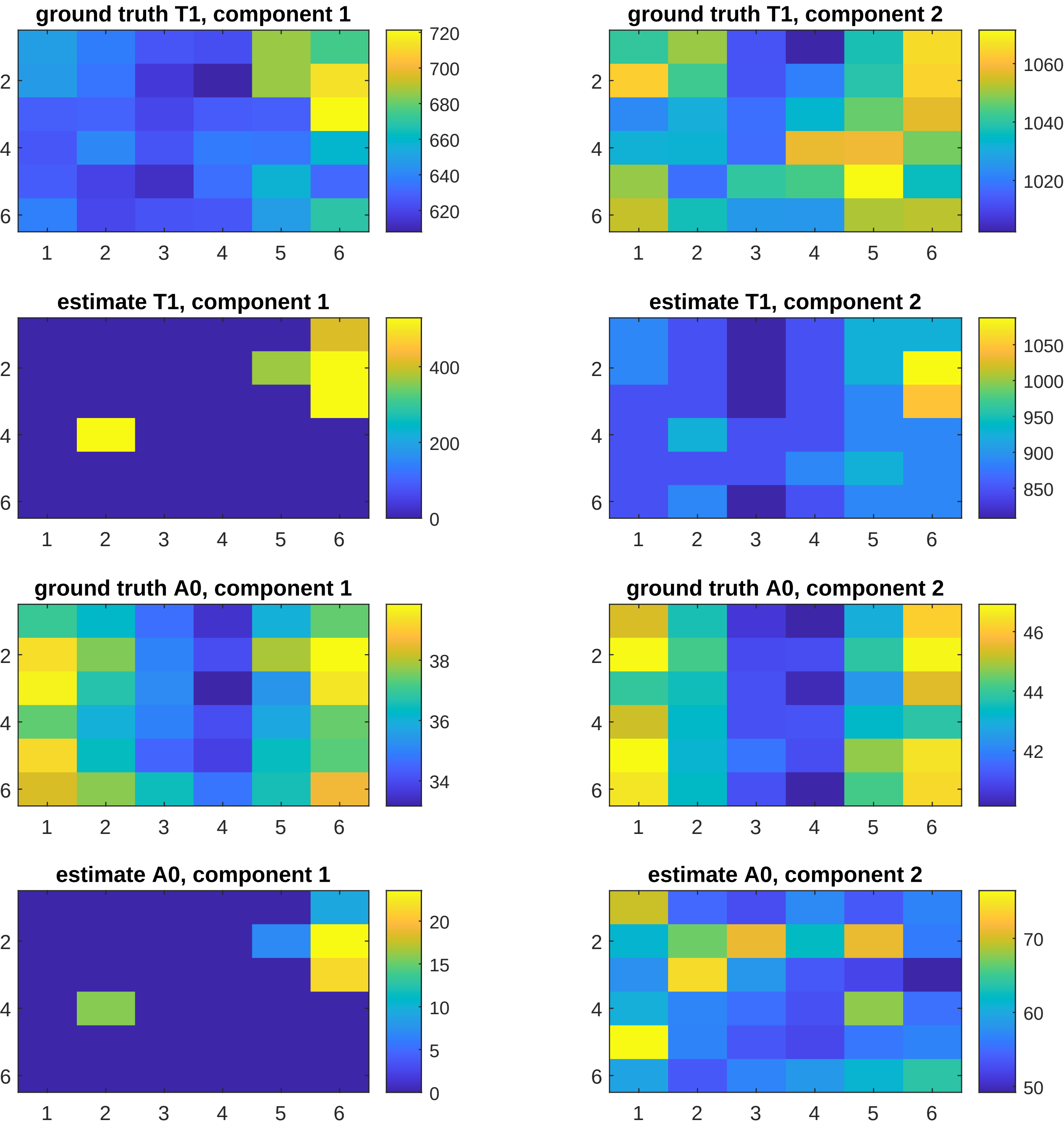
ground truth T1, component 1
ground truth T1, component 2
estimate T1, component 1
estimate T1, component 2
ground truth A0, component 1
ground truth A0, component 2
estimate A0, component 1
estimate A0, component 2

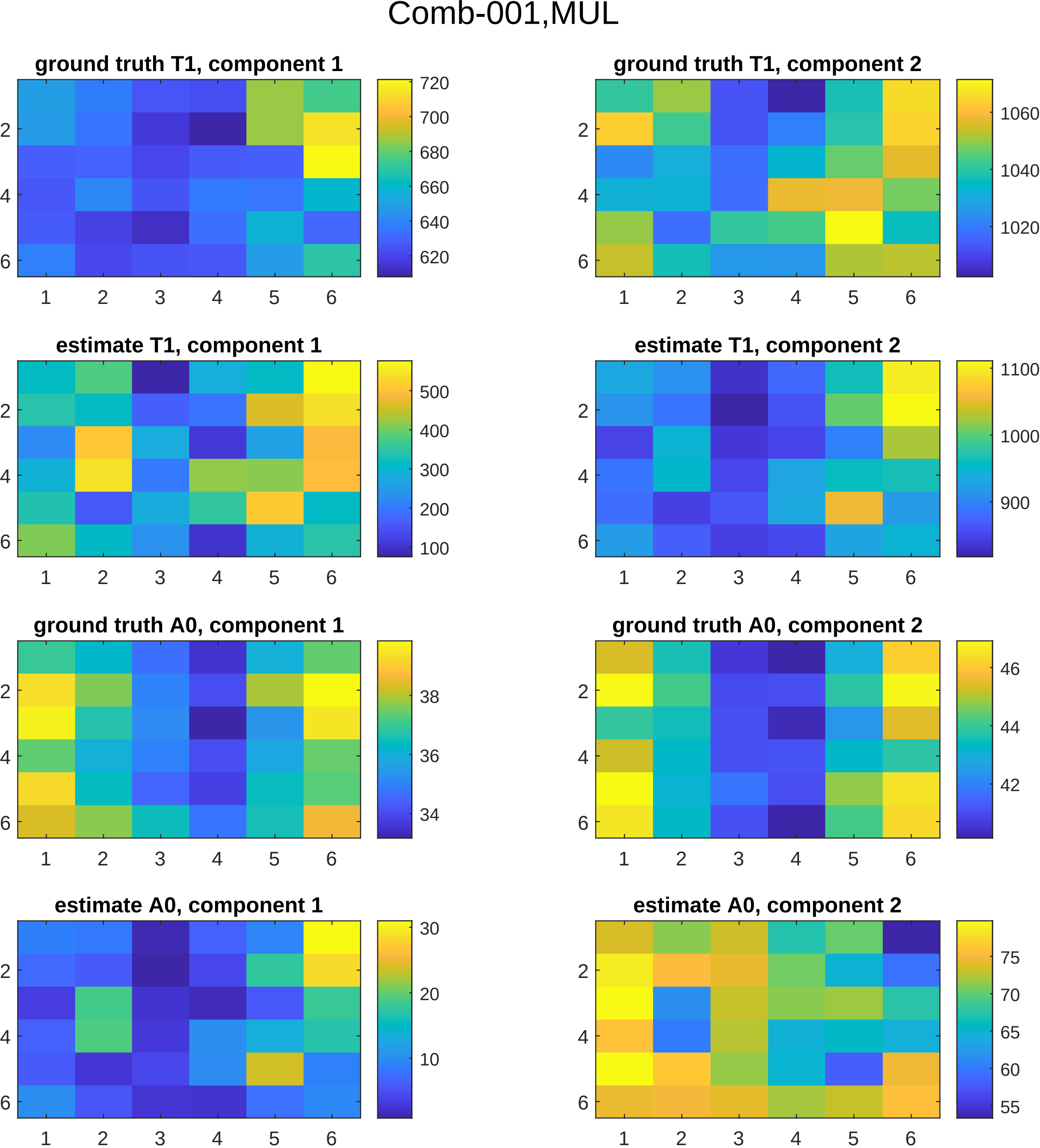
Comb-001,MUL
ground truth T1, component 1
720
700
680
660
640
620
ground truth T1, component 2
1060
1040
1020
estimate T1, component 1
500
400
300
200
100
estimate T1, component 2
1100
1000
900
ground truth A0, component 1
38
36
34
ground truth A0, component 2
46
44
42
estimate A0, component 1
30
20
10
estimate A0, component 2
75
70
65
60
55
1
2
3
4
5
6

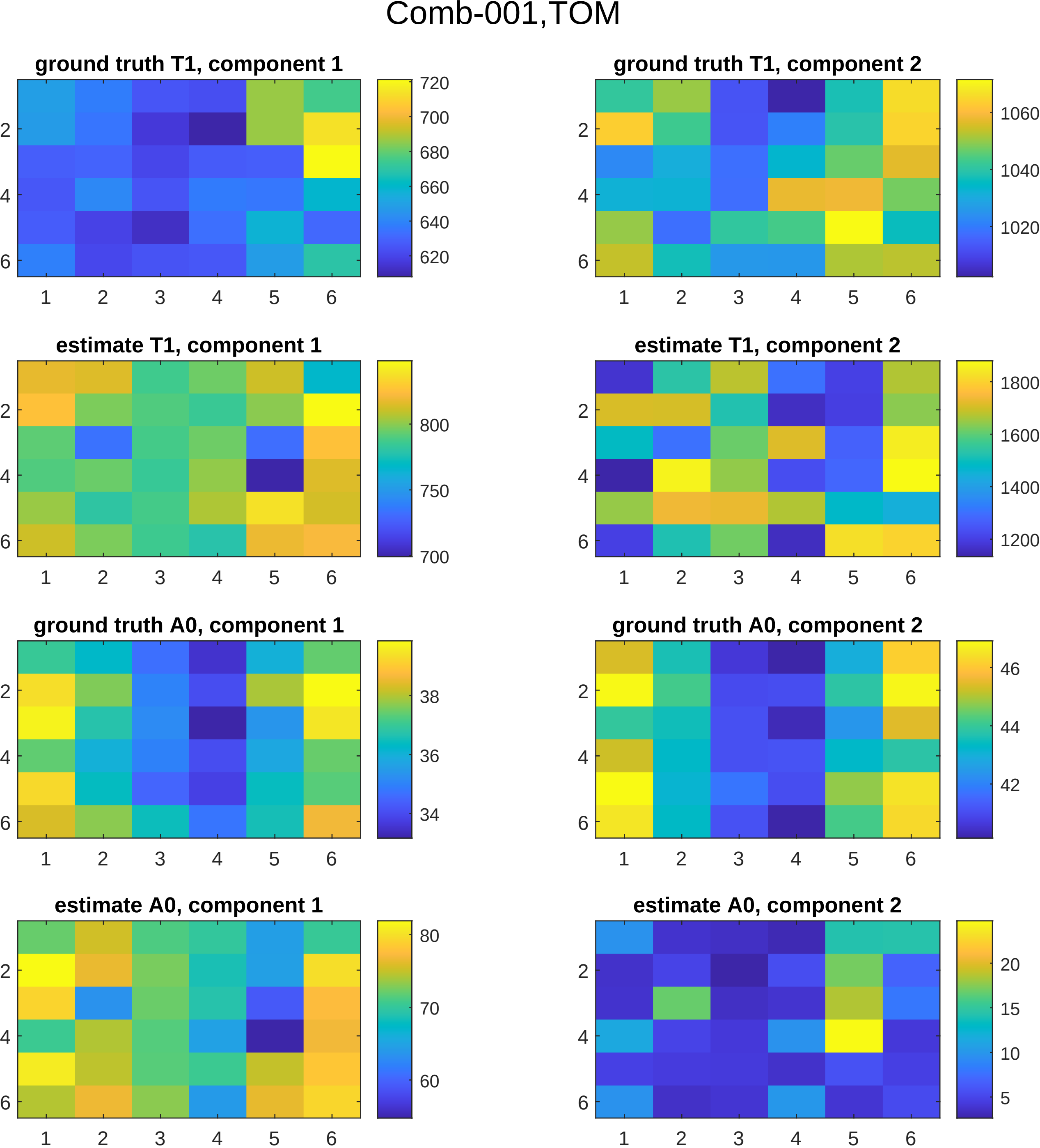

Comb-001,TOM
ground truth T1, component 1
ground truth T1, component 2
estimate T1, component 1
estimate T1, component 2
ground truth A0, component 1
ground truth A0, component 2
estimate A0, component 1
estimate A0, component 2

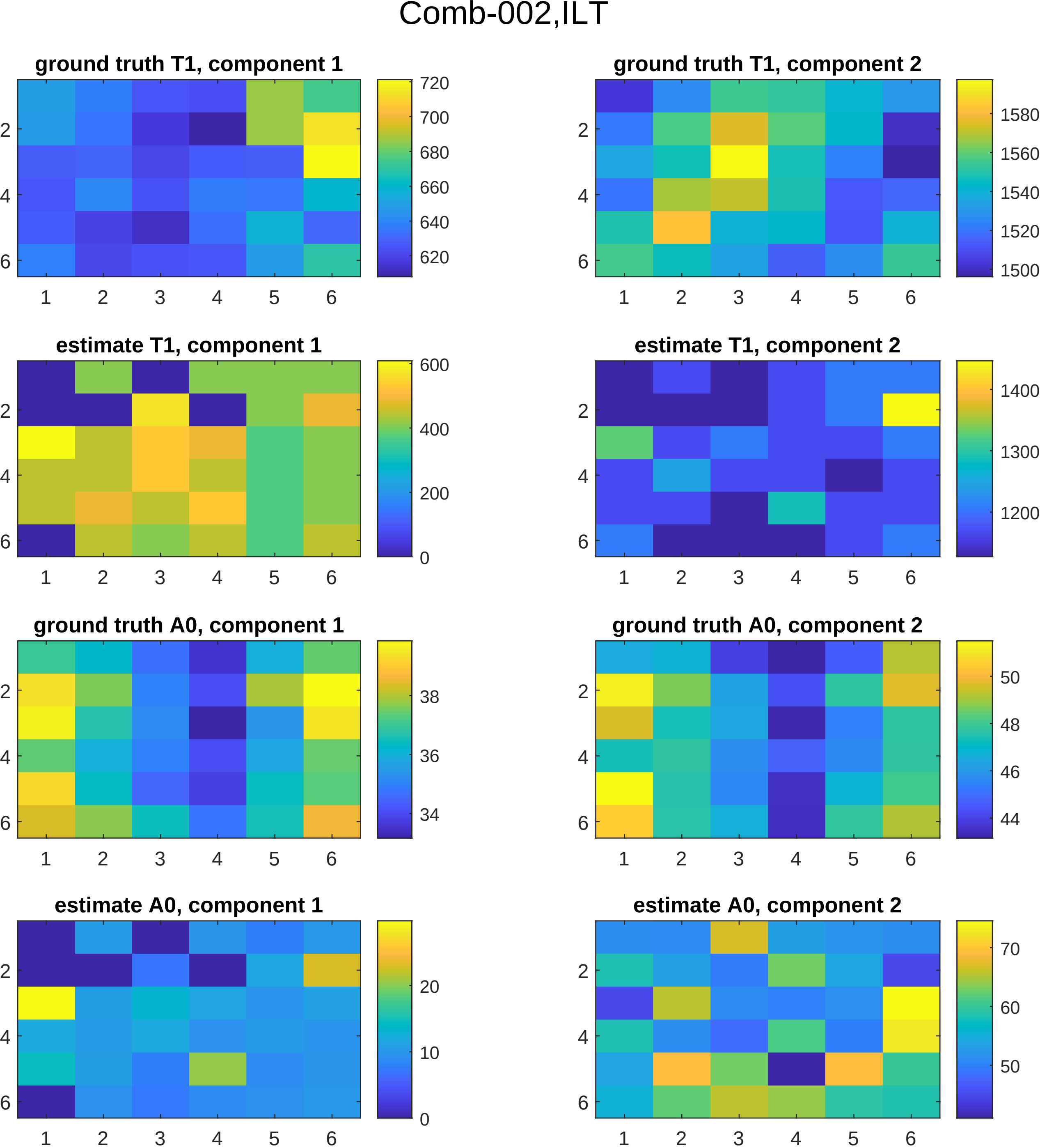
Comb-002,ILT
ground truth T1, component 1
ground truth T1, component 2
estimate T1, component 1
estimate T1, component 2
ground truth A0, component 1
ground truth A0, component 2
estimate A0, component 1
estimate A0, component 2

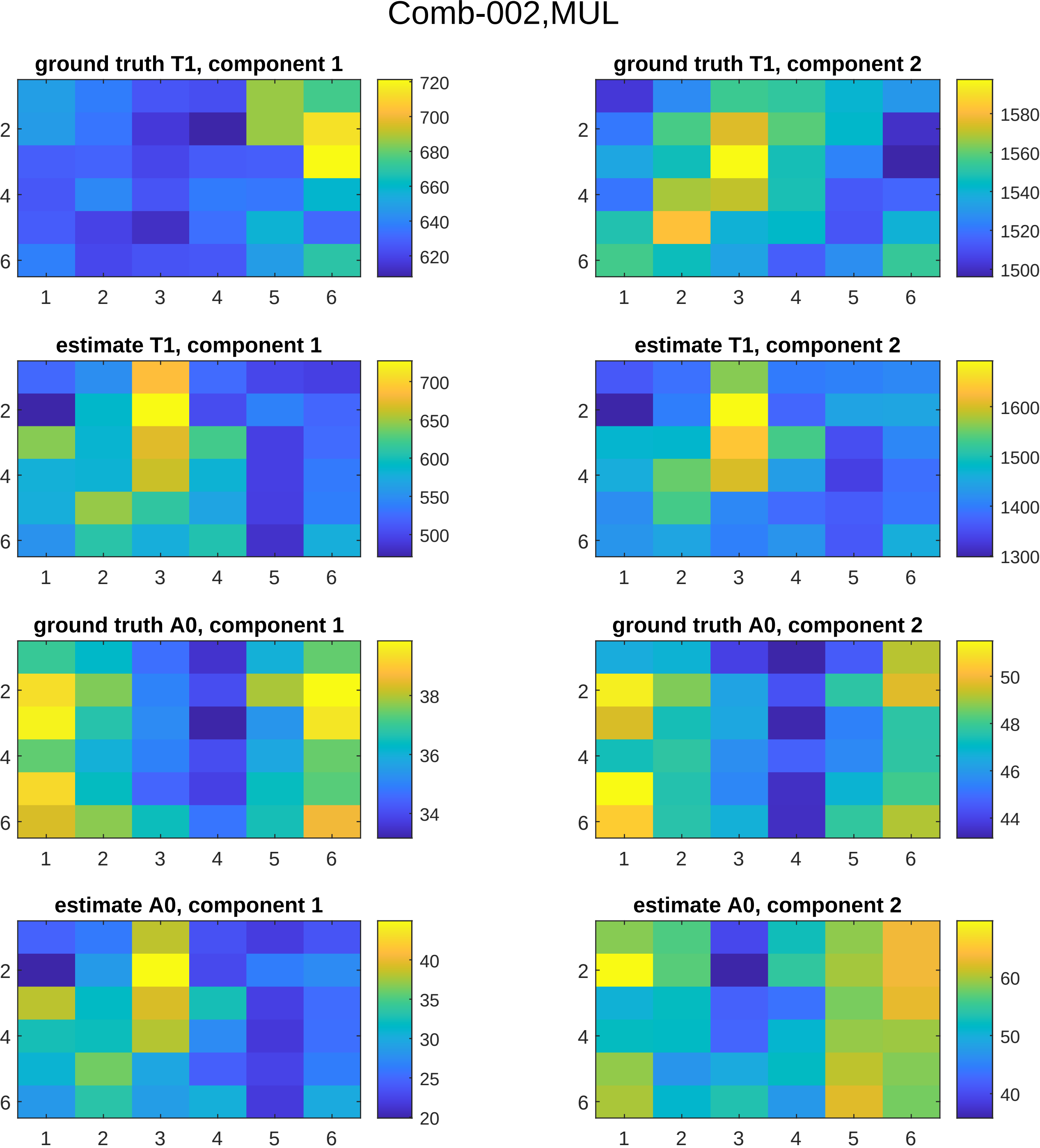

Comb-002,MUL
ground truth T1, component 1
ground truth T1, component 2
estimate T1, component 1
estimate T1, component 2
ground truth A0, component 1
ground truth A0, component 2
estimate A0, component 1
estimate A0, component 2

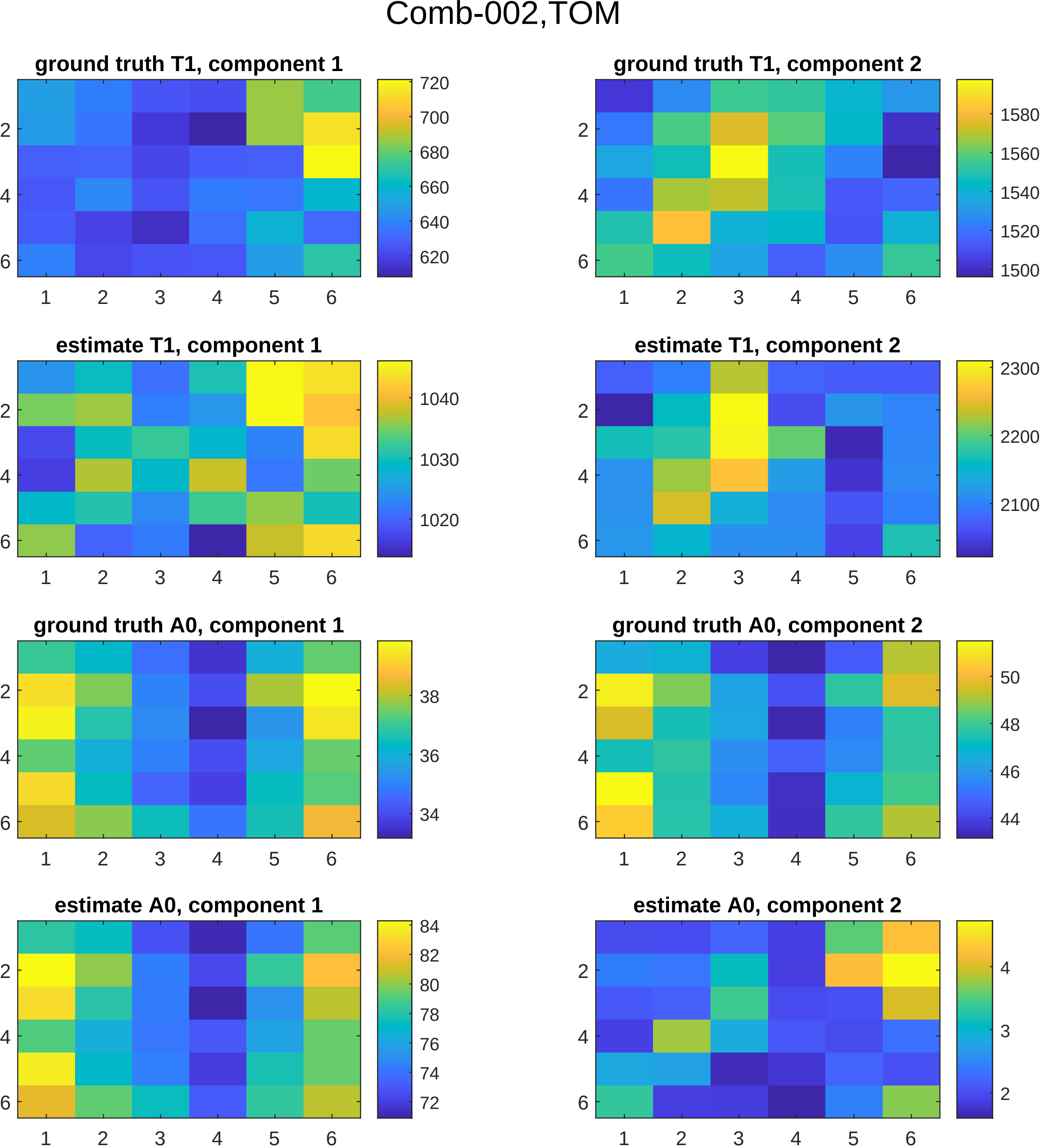

Comb-002,TOM
ground truth T1, component 1
ground truth T1, component 2
estimate T1, component 1
estimate T1, component 2
ground truth A0, component 1
ground truth A0, component 2
estimate A0, component 1
estimate A0, component 2

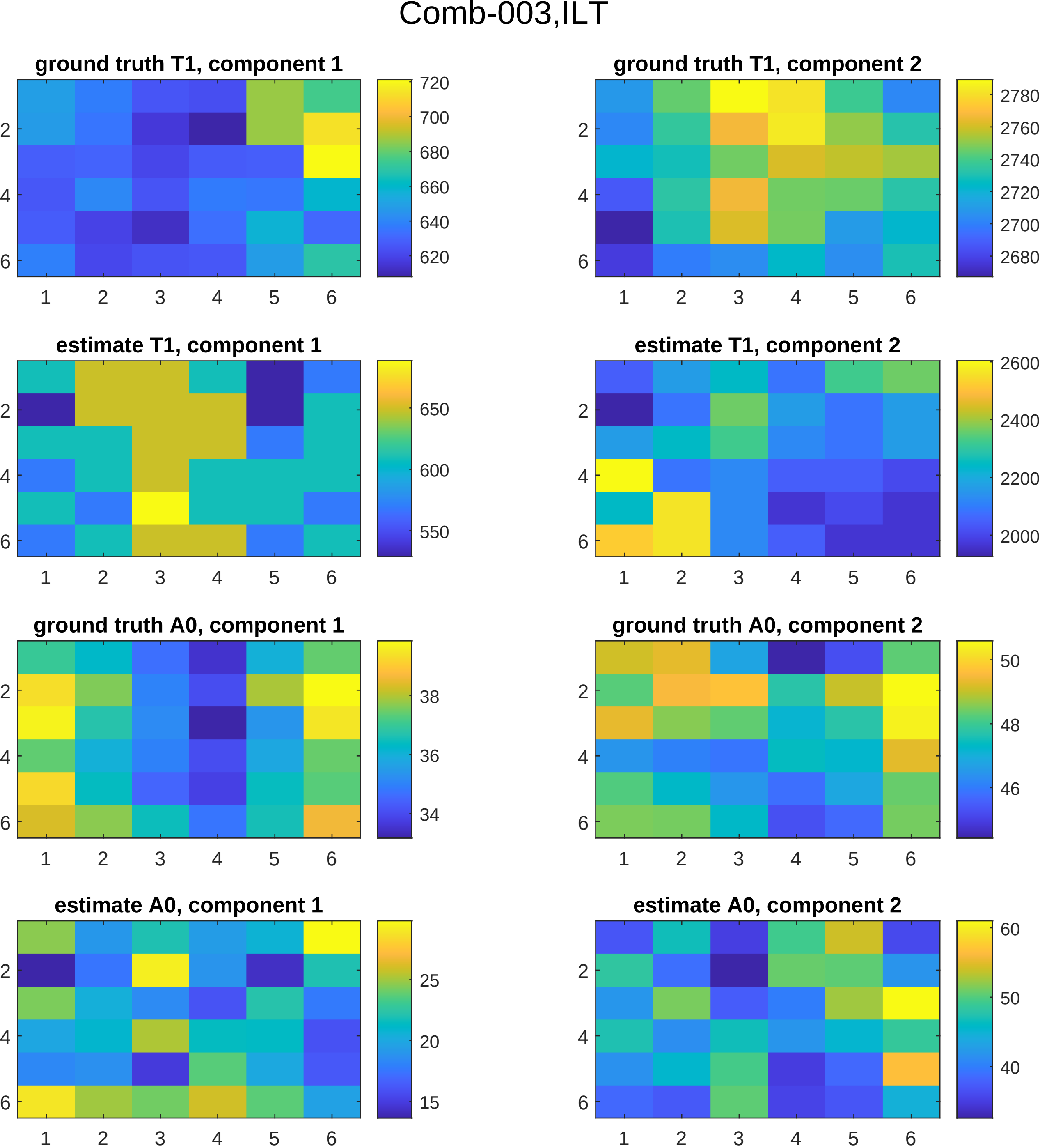

Comb-003,ILT
ground truth T1, component 1
ground truth T1, component 2
estimate T1, component 1
estimate T1, component 2
ground truth A0, component 1
ground truth A0, component 2
estimate A0, component 1
estimate A0, component 2

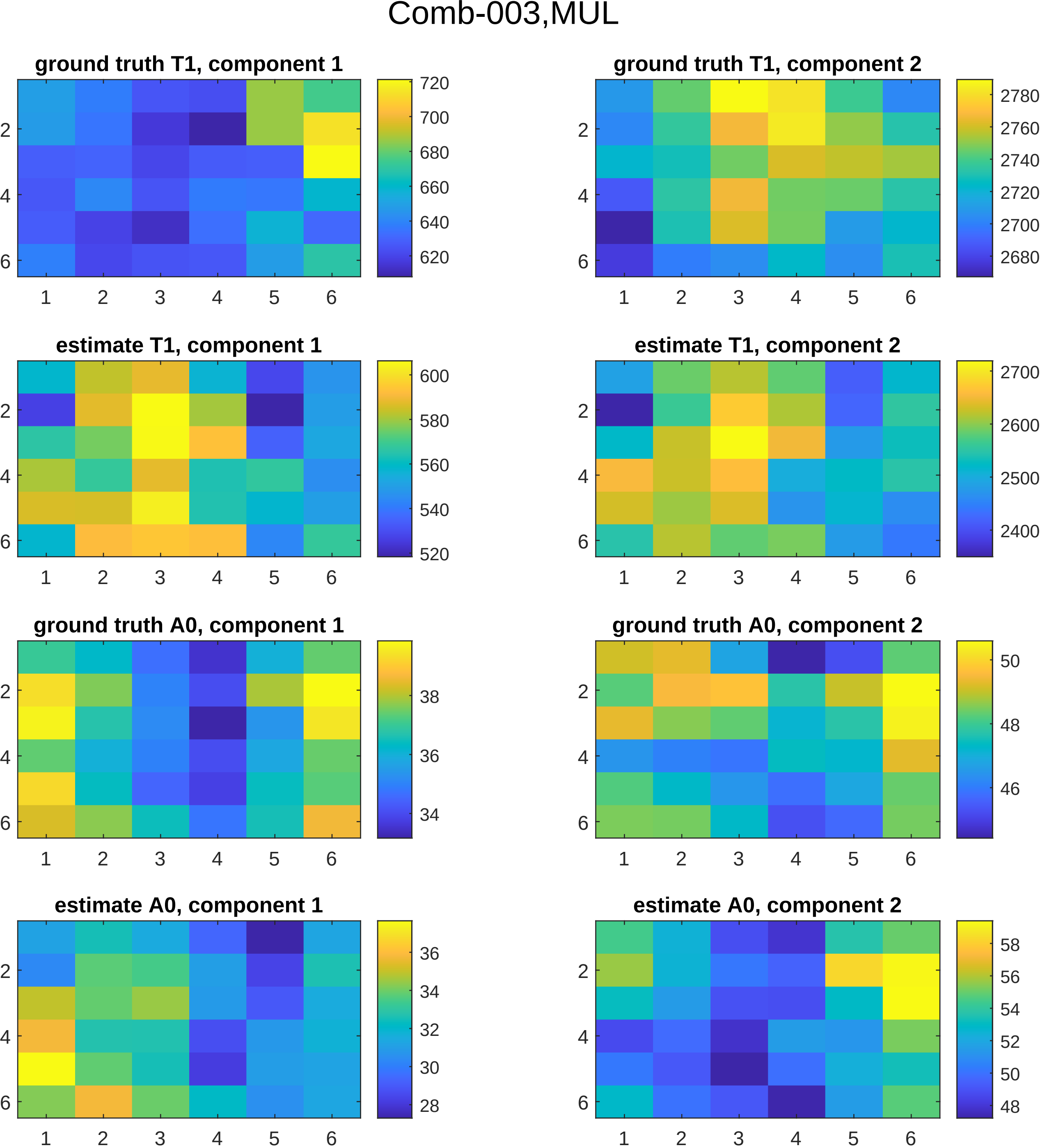
Comb-003,MUL
ground truth T1, component 1
ground truth T1, component 2
estimate T1, component 1
estimate T1, component 2
ground truth A0, component 1
ground truth A0, component 2
estimate A0, component 1
estimate A0, component 2

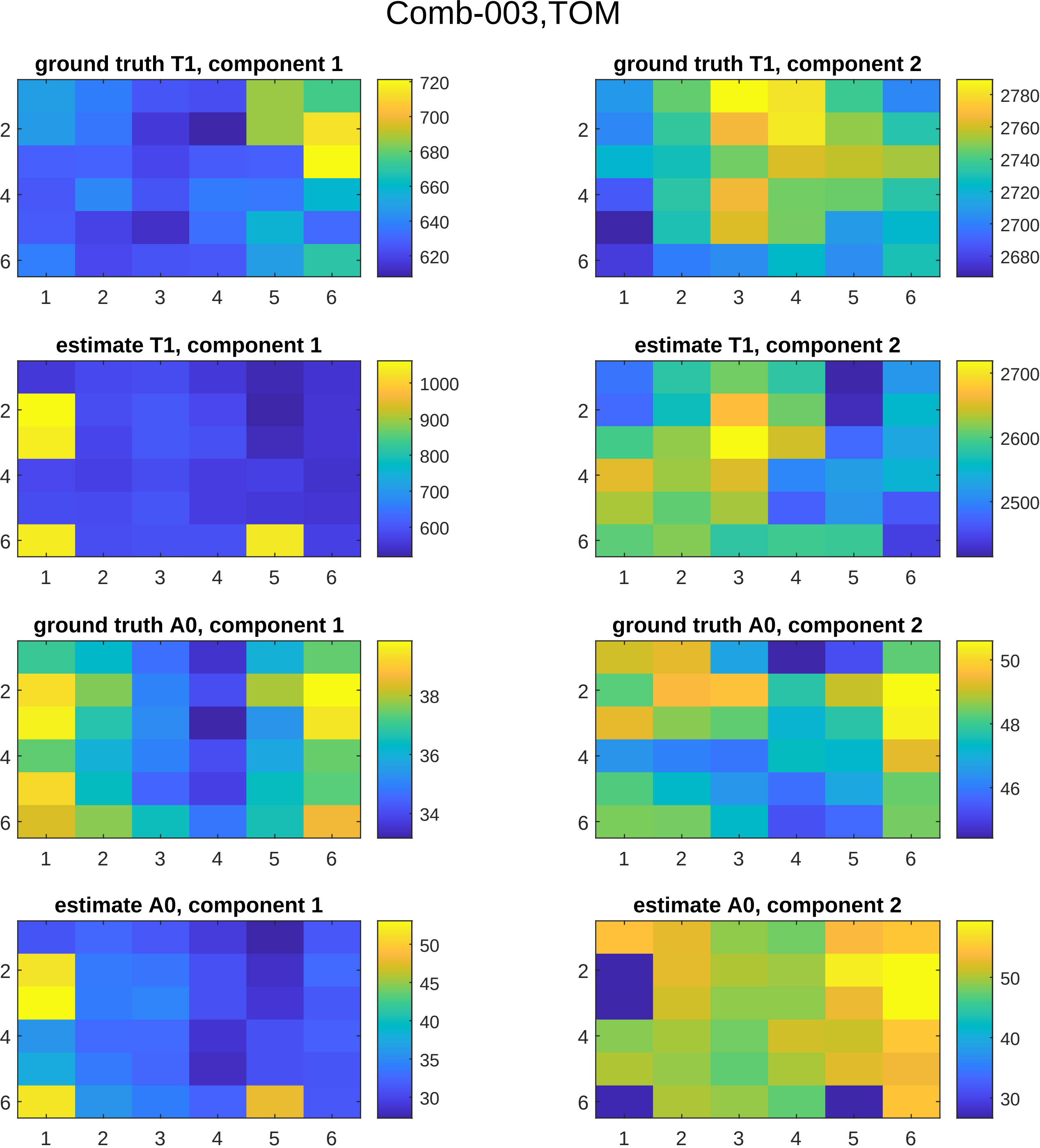
Comb-003,TOM
ground truth T1, component 1
ground truth T1, component 2
estimate T1, component 1
estimate T1, component 2
ground truth A0, component 1
ground truth A0, component 2
estimate A0, component 1
estimate A0, component 2

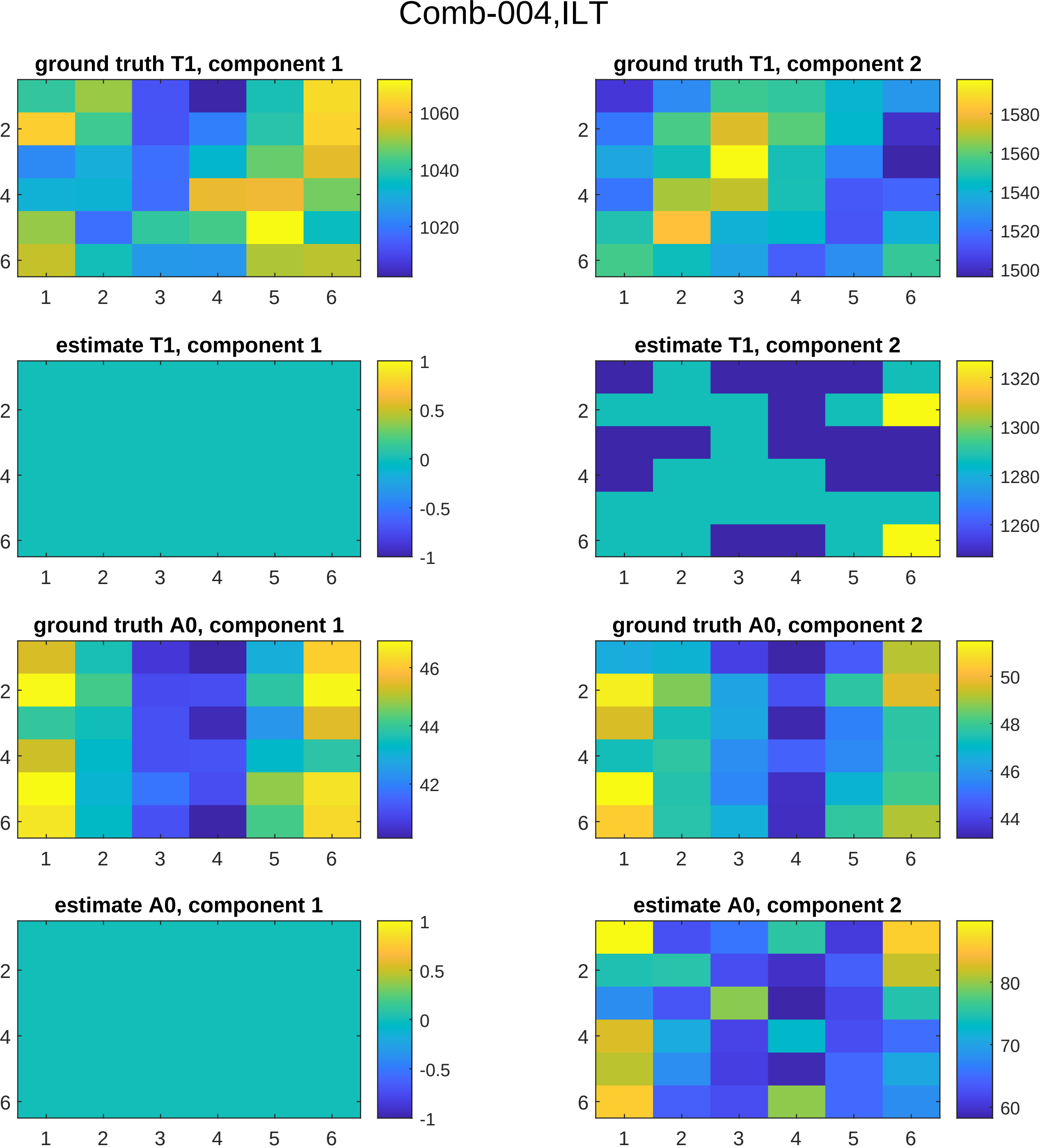
Comb-004,ILT
ground truth T1, component 1
ground truth T1, component 2
estimate T1, component 1
estimate T1, component 2
ground truth A0, component 1
ground truth A0, component 2
estimate A0, component 1
estimate A0, component 2

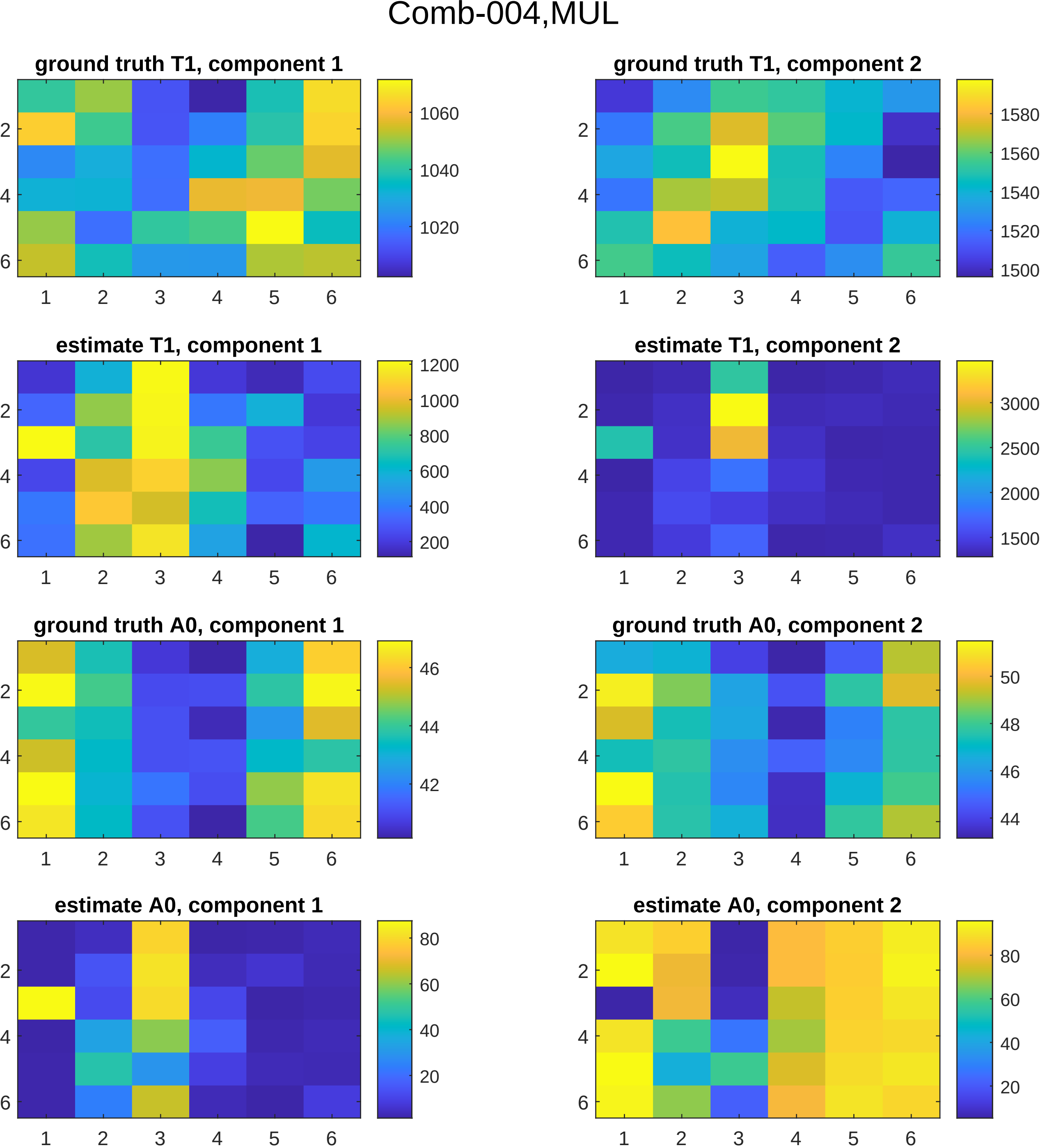
Comb-004,MUL
ground truth T1, component 1
ground truth T1, component 2
estimate T1, component 1
estimate T1, component 2
ground truth A0, component 1
ground truth A0, component 2
estimate A0, component 1
estimate A0, component 2

Comb-004,TOM

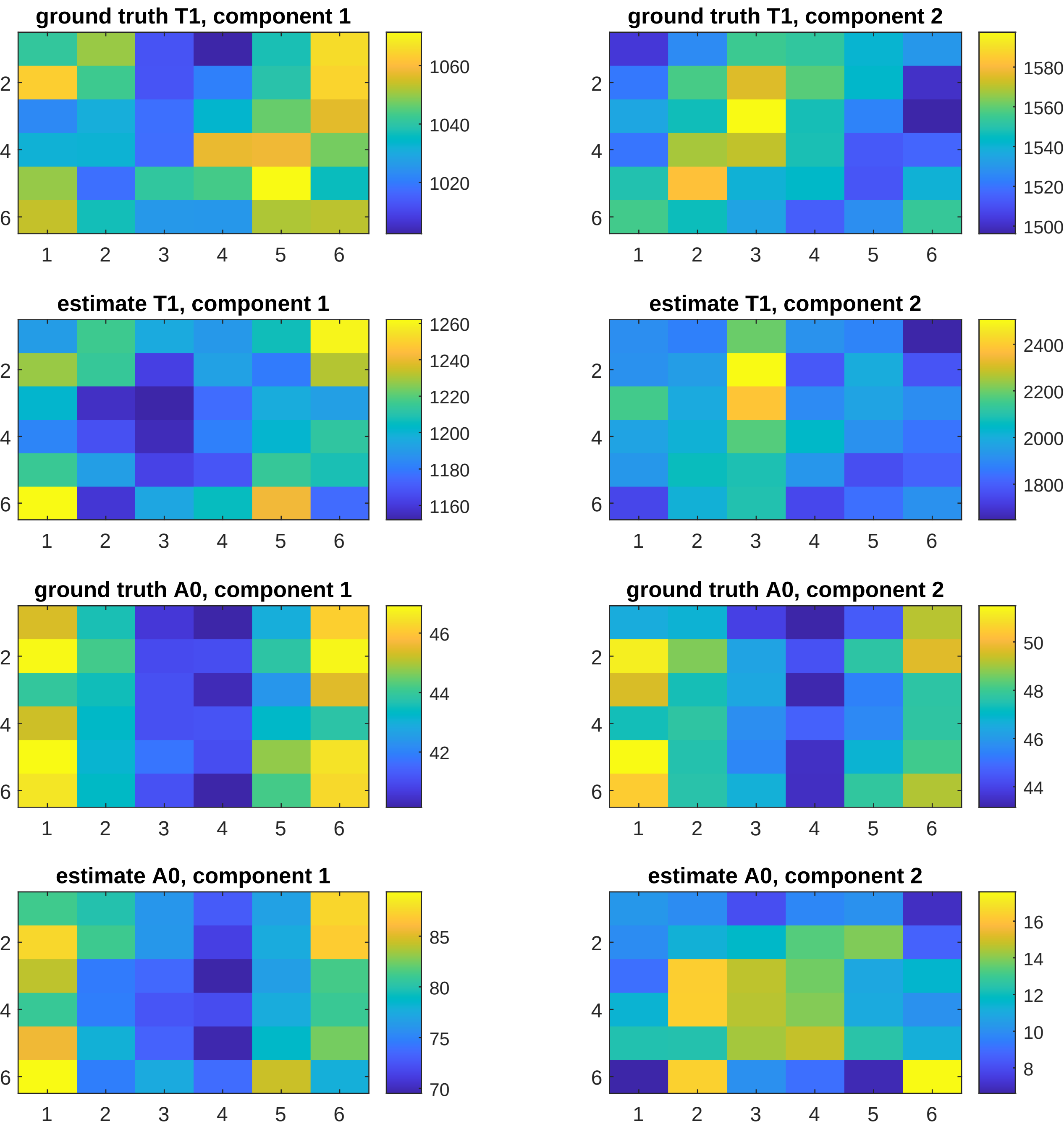
ground truth T1, component 1
ground truth T1, component 2
estimate T1, component 1
estimate T1, component 2
ground truth A0, component 1
ground truth A0, component 2
estimate A0, component 1
estimate A0, component 2

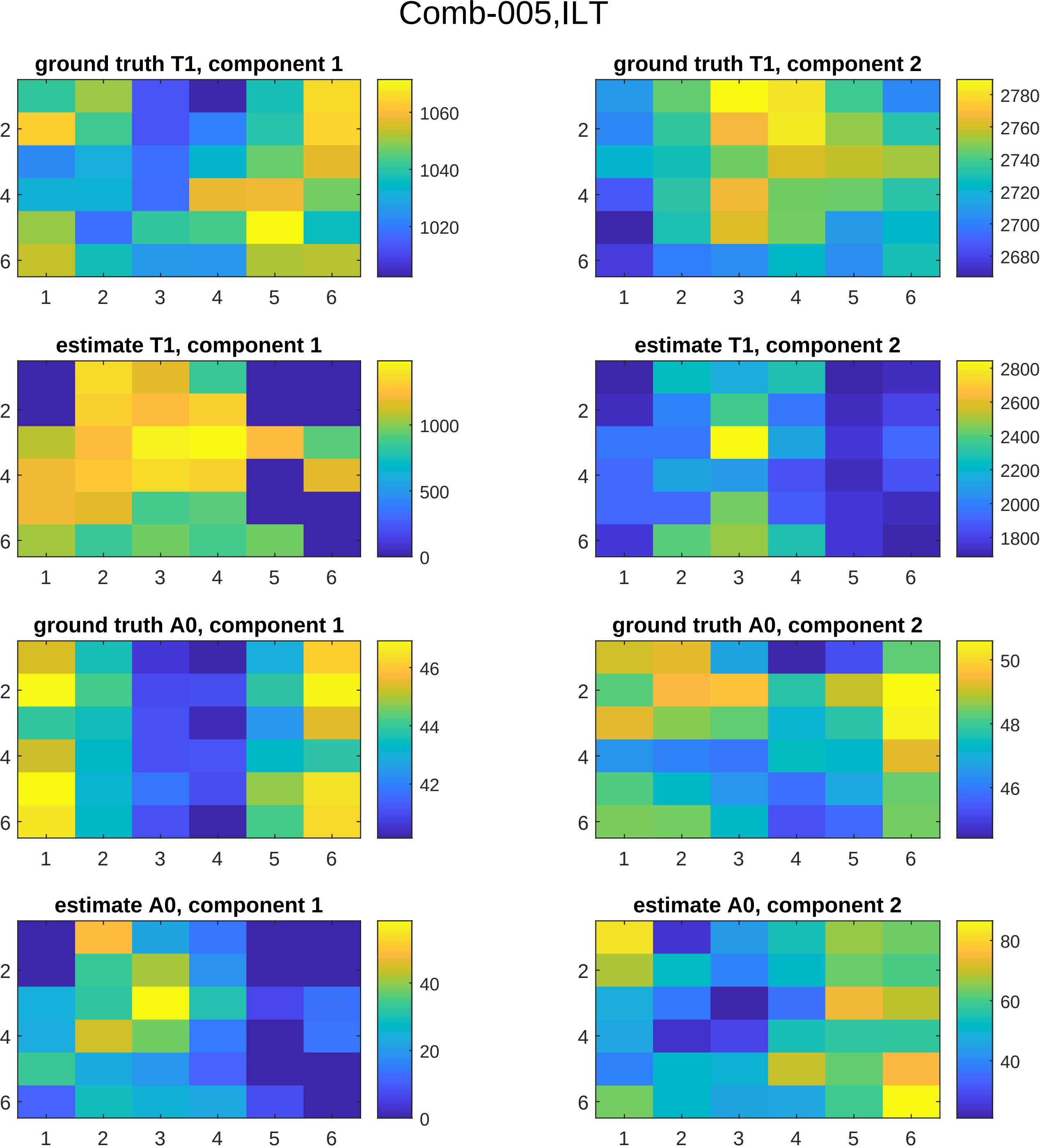
Comb-005,ILT
ground truth T1, component 1
ground truth T1, component 2
estimate T1, component 1
estimate T1, component 2
ground truth A0, component 1
ground truth A0, component 2
estimate A0, component 1
estimate A0, component 2
1060
1040
1020
2780
2760
2740
2720
2700
2680
1000
500
0
2800
2600
2400
2200
2000
1800
46
44
42
50
48
46
40
20
0
80
60
40
2
4
6
1
2
3
4
5
6

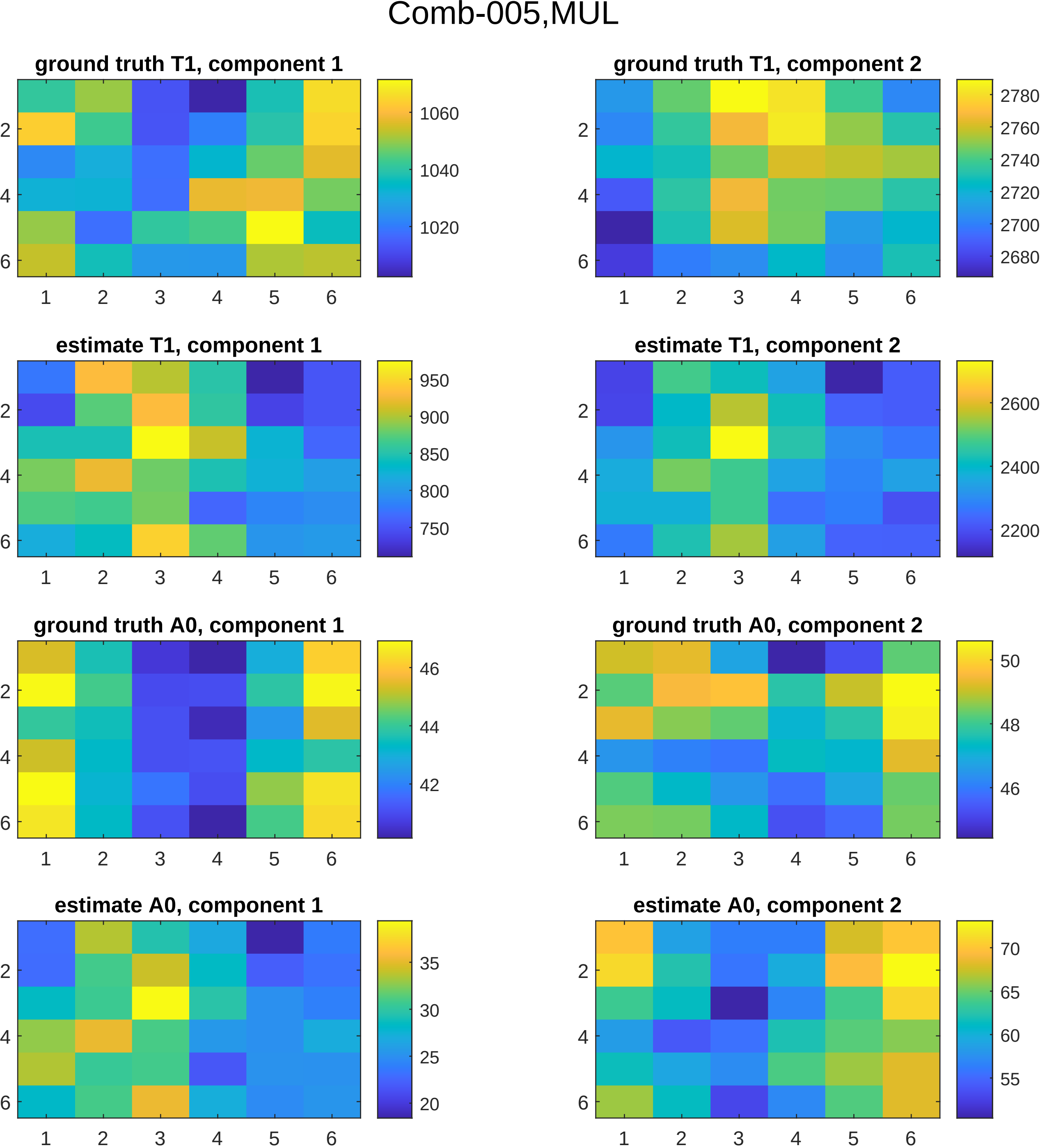
Comb-005,MUL
ground truth T1, component 1
ground truth T1, component 2
estimate T1, component 1
estimate T1, component 2
ground truth A0, component 1
ground truth A0, component 2
estimate A0, component 1
estimate A0, component 2

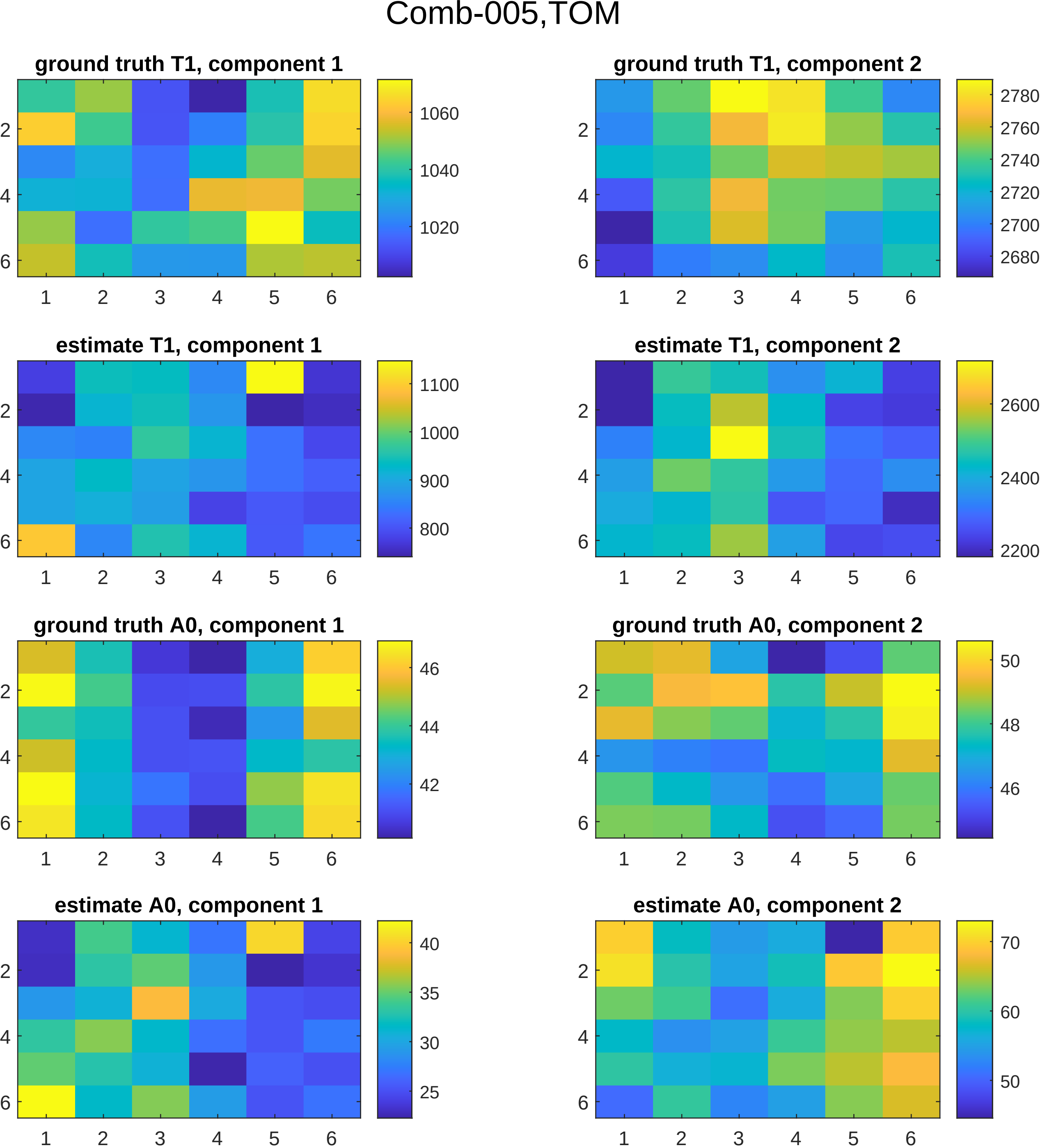
Comb-005,TOM
ground truth T1, component 1
ground truth T1, component 2
estimate T1, component 1
estimate T1, component 2
ground truth A0, component 1
ground truth A0, component 2
estimate A0, component 1
estimate A0, component 2
1060
1040
1020
2780
2760
2740
2720
2700
2680
1100
1000
900
800
2600
2400
2200
46
44
42
50
48
46
40
35
30
25
70
60
50

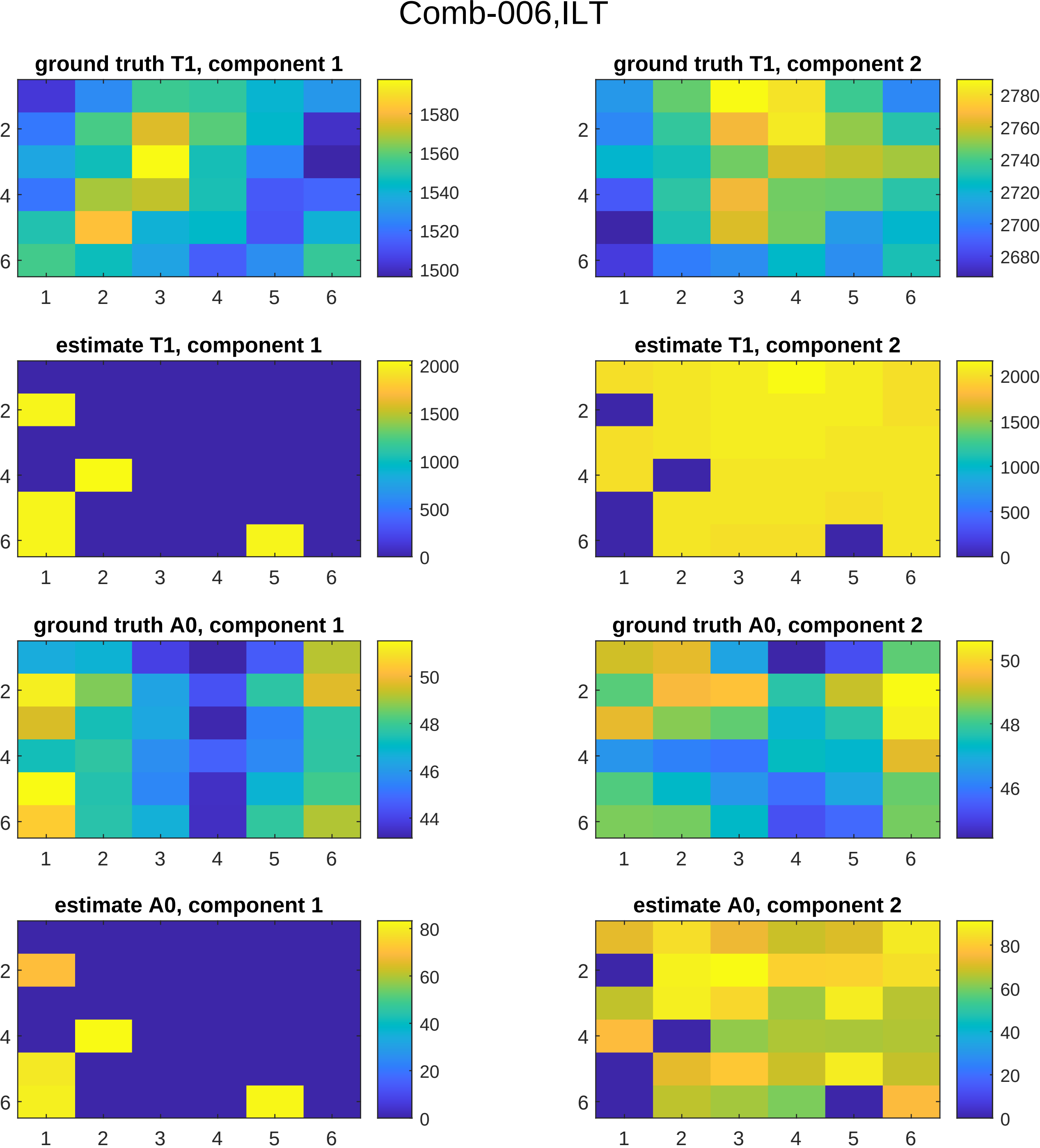
Comb-006,ILT
ground truth T1, component 1
ground truth T1, component 2
estimate T1, component 1
estimate T1, component 2
ground truth A0, component 1
ground truth A0, component 2
estimate A0, component 1
estimate A0, component 2

Comb-006,MUL

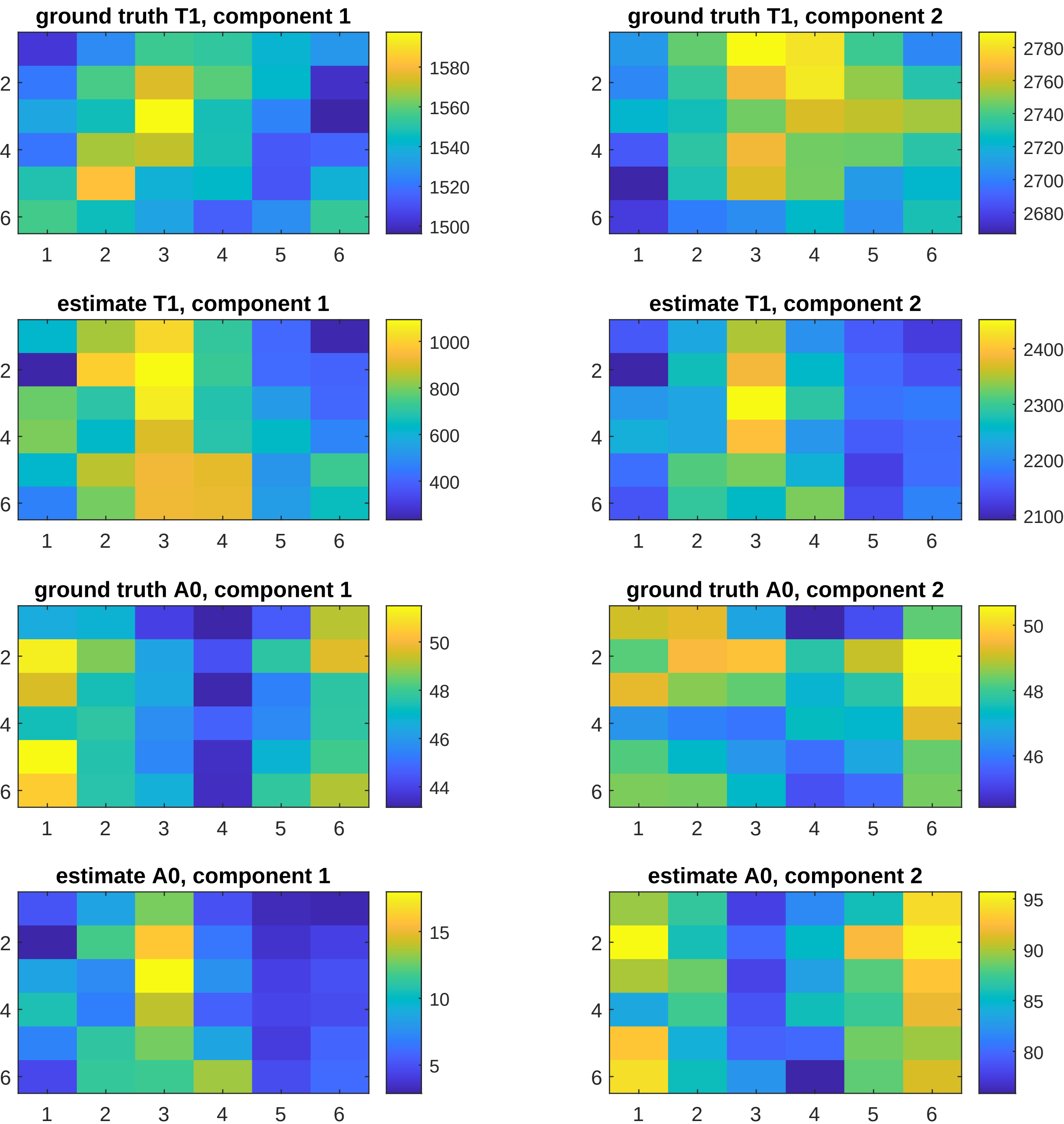
ground truth T1, component 1
1580
1560
1540
1520
1500
ground truth T1, component 2
2780
2760
2740
2720
2700
2680
estimate T1, component 1
1000
800
600
400
estimate T1, component 2
2400
2300
2200
2100
ground truth A0, component 1
50
48
46
44
ground truth A0, component 2
50
48
46
estimate A0, component 1
15
10
5
estimate A0, component 2
95
90
85
80

Comb-006,TOM

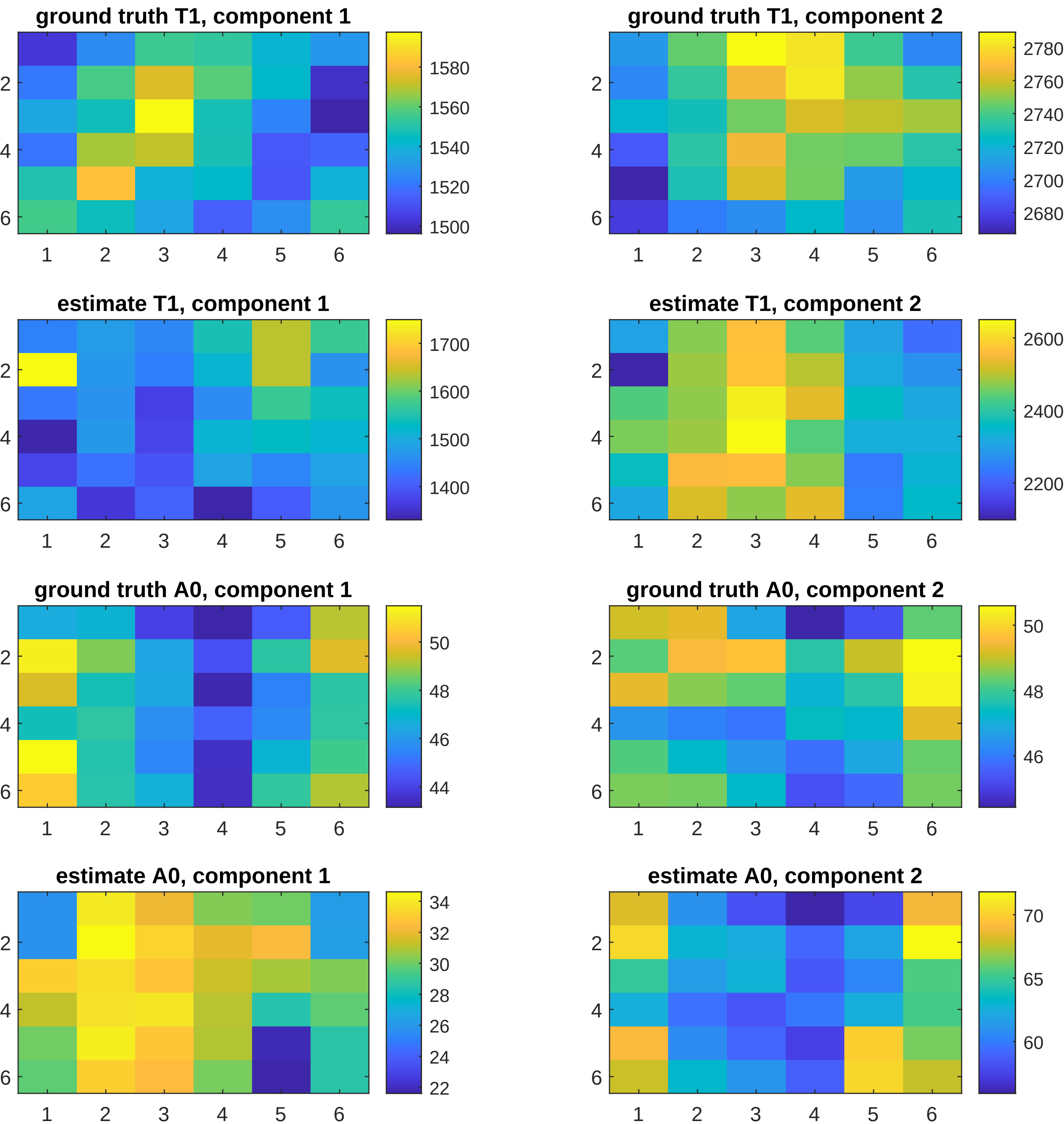
ground truth T1, component 1
ground truth T1, component 2
estimate T1, component 1
estimate T1, component 2
ground truth A0, component 1
ground truth A0, component 2
estimate A0, component 1
estimate A0, component 2

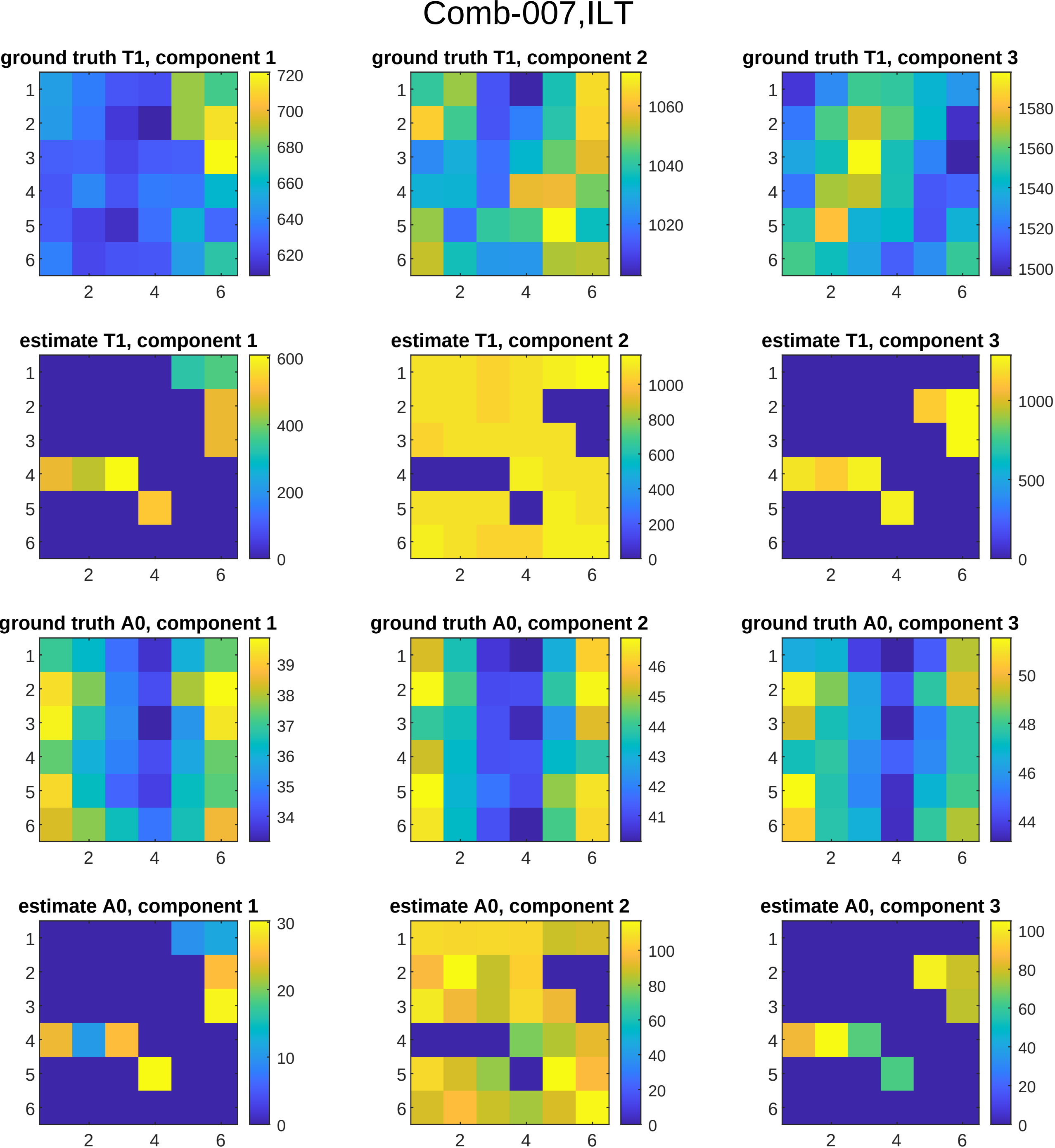

Comb-007,ILT
ground truth T1, component 1
ground truth T1, component 2
ground truth T1, component 3
estimate T1, component 1
estimate T1, component 2
estimate T1, component 3
ground truth A0, component 1
ground truth A0, component 2
ground truth A0, component 3
estimate A0, component 1
estimate A0, component 2
estimate A0, component 3

Comb-007,MUL

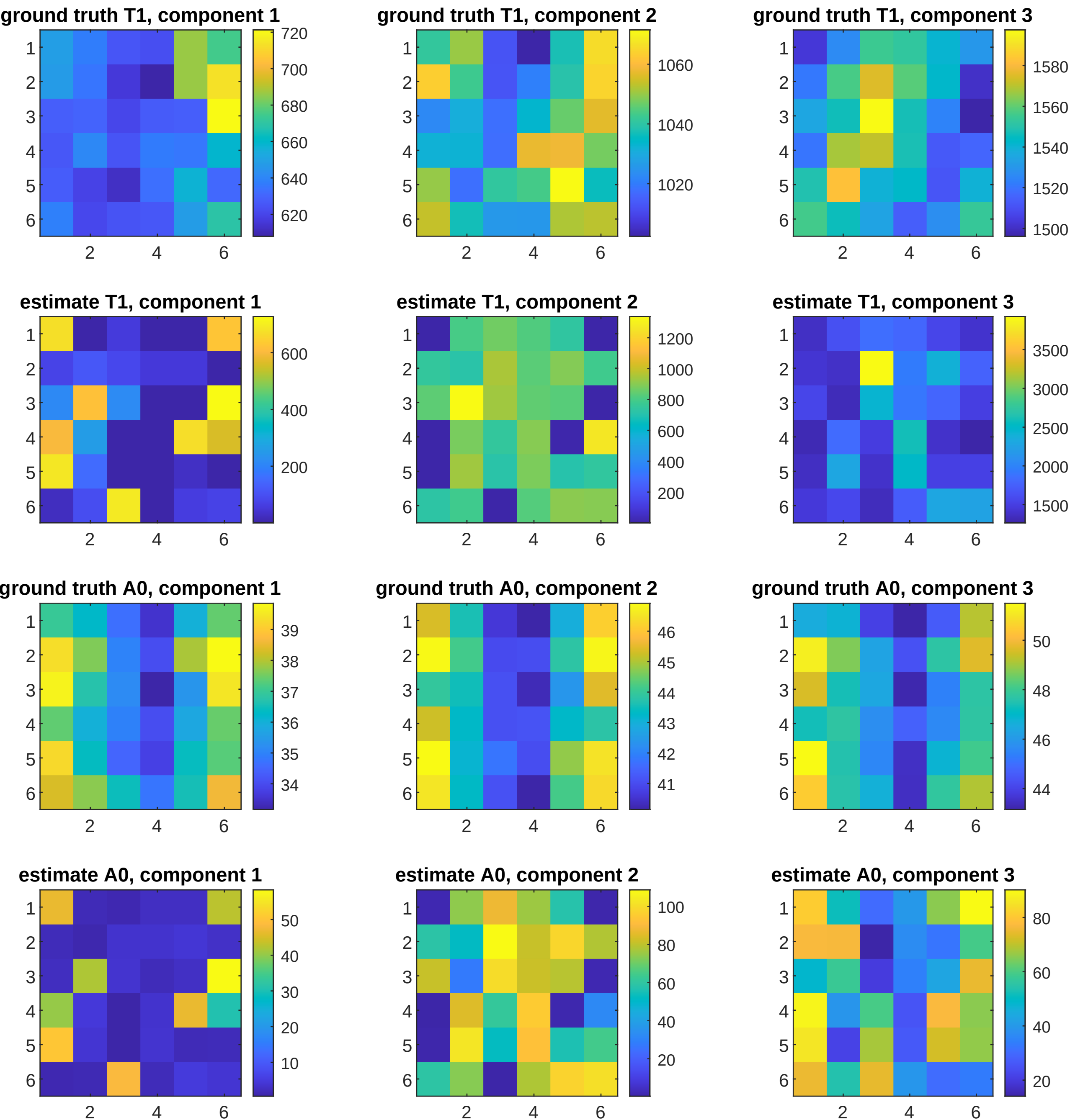
ground truth T1, component 1
720
700
680
660
640
620
ground truth T1, component 2
1060
1040
1020
ground truth T1, component 3
1580
1560
1540
1520
1500
estimate T1, component 1
600
400
200
estimate T1, component 2
1200
1000
800
600
400
200
estimate T1, component 3
3500
3000
2500
2000
1500
ground truth A0, component 1
39
38
37
36
35
34
ground truth A0, component 2
46
45
44
43
42
41
ground truth A0, component 3
50
48
46
44
estimate A0, component 1
50
40
30
20
10
estimate A0, component 2
100
80
60
40
20
estimate A0, component 3
80
60
40
20
1
2
3
4
5
6
2
4
6

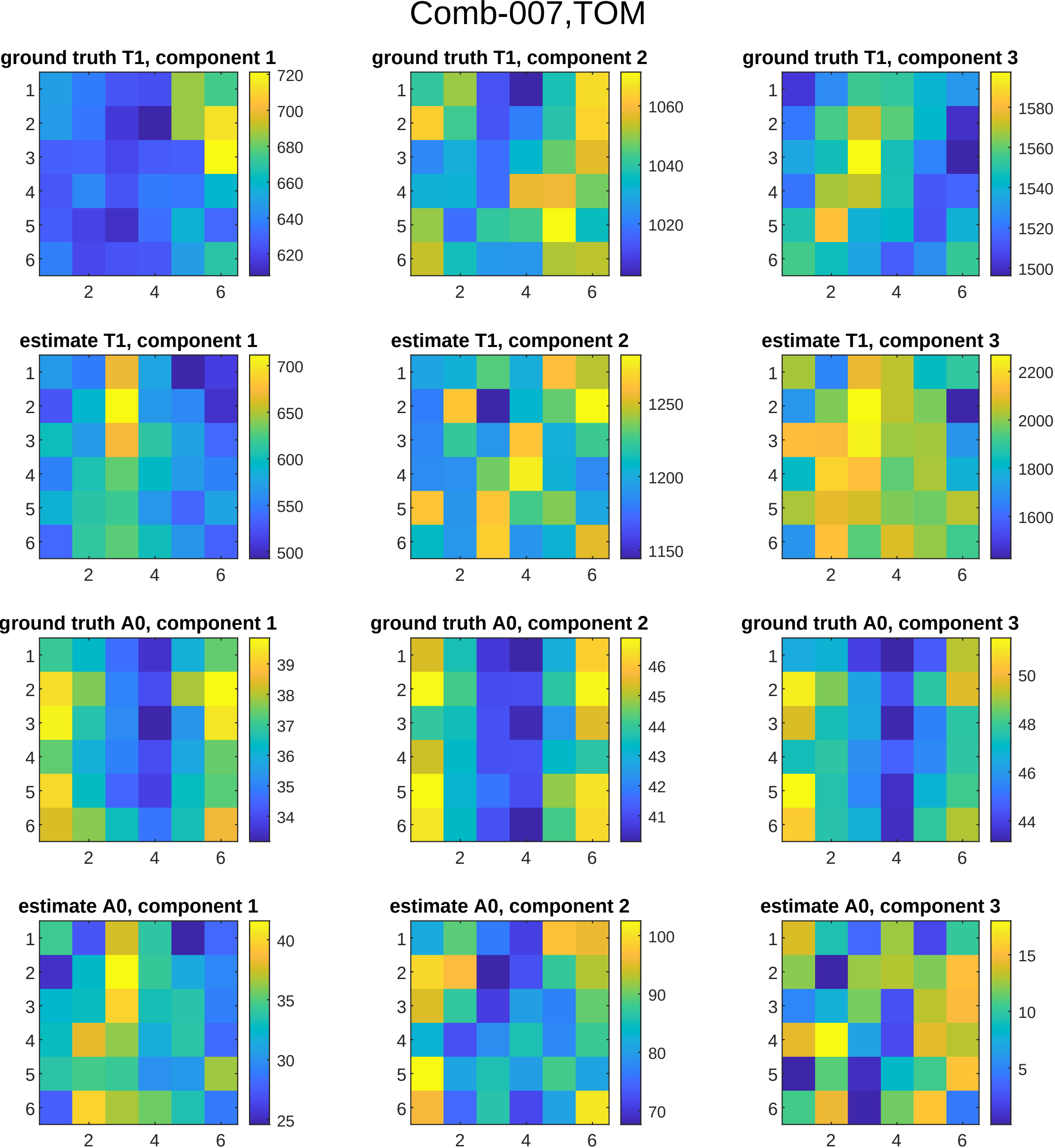
Comb-007,TOM
ground truth T1, component 1
ground truth T1, component 2
ground truth T1, component 3
estimate T1, component 1
estimate T1, component 2
estimate T1, component 3
ground truth A0, component 1
ground truth A0, component 2
ground truth A0, component 3
estimate A0, component 1
estimate A0, component 2
estimate A0, component 3

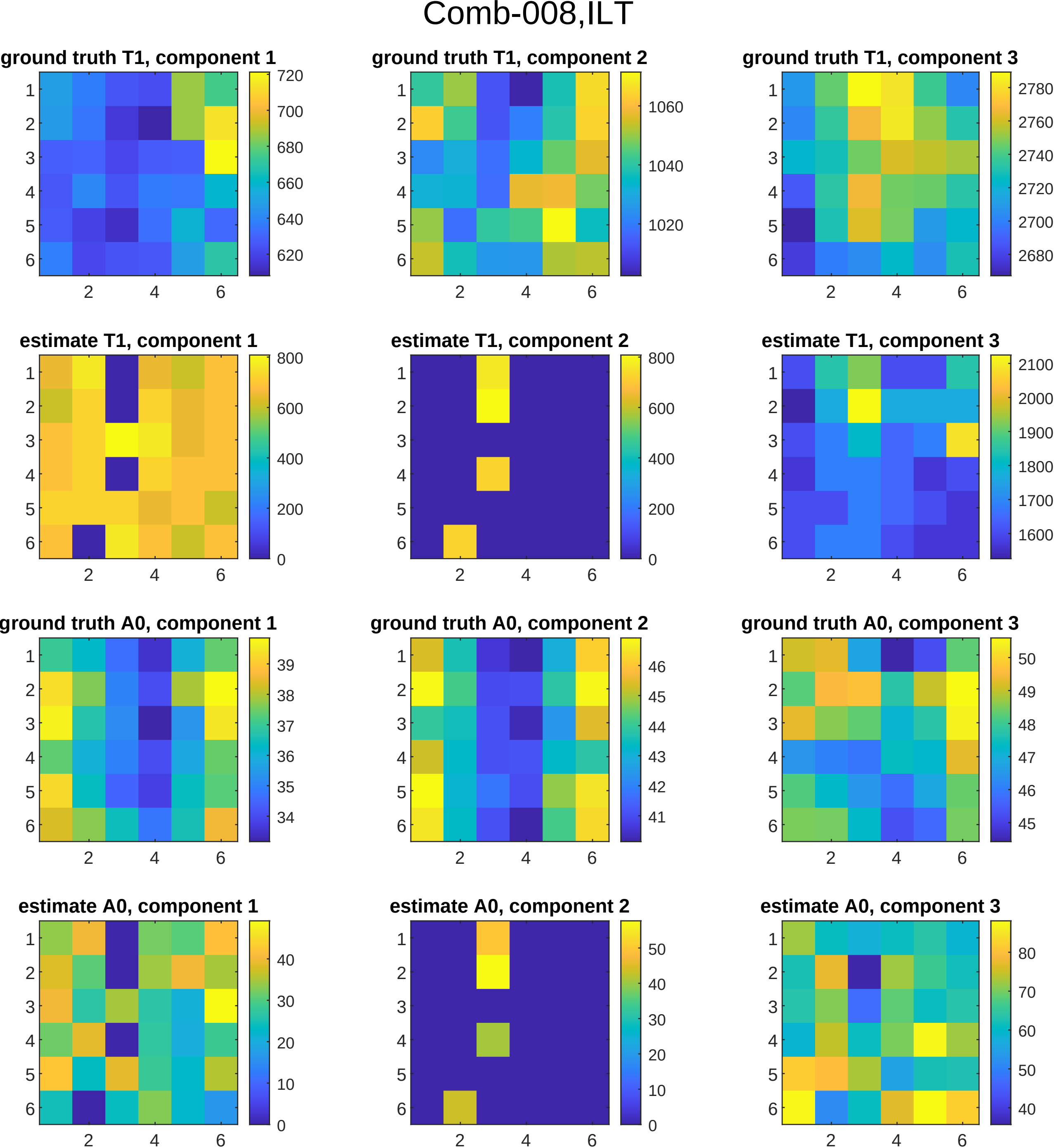
Comb-008,ILT
ground truth T1, component 1
ground truth T1, component 2
ground truth T1, component 3
estimate T1, component 1
estimate T1, component 2
estimate T1, component 3
ground truth A0, component 1
ground truth A0, component 2
ground truth A0, component 3
estimate A0, component 1
estimate A0, component 2
estimate A0, component 3

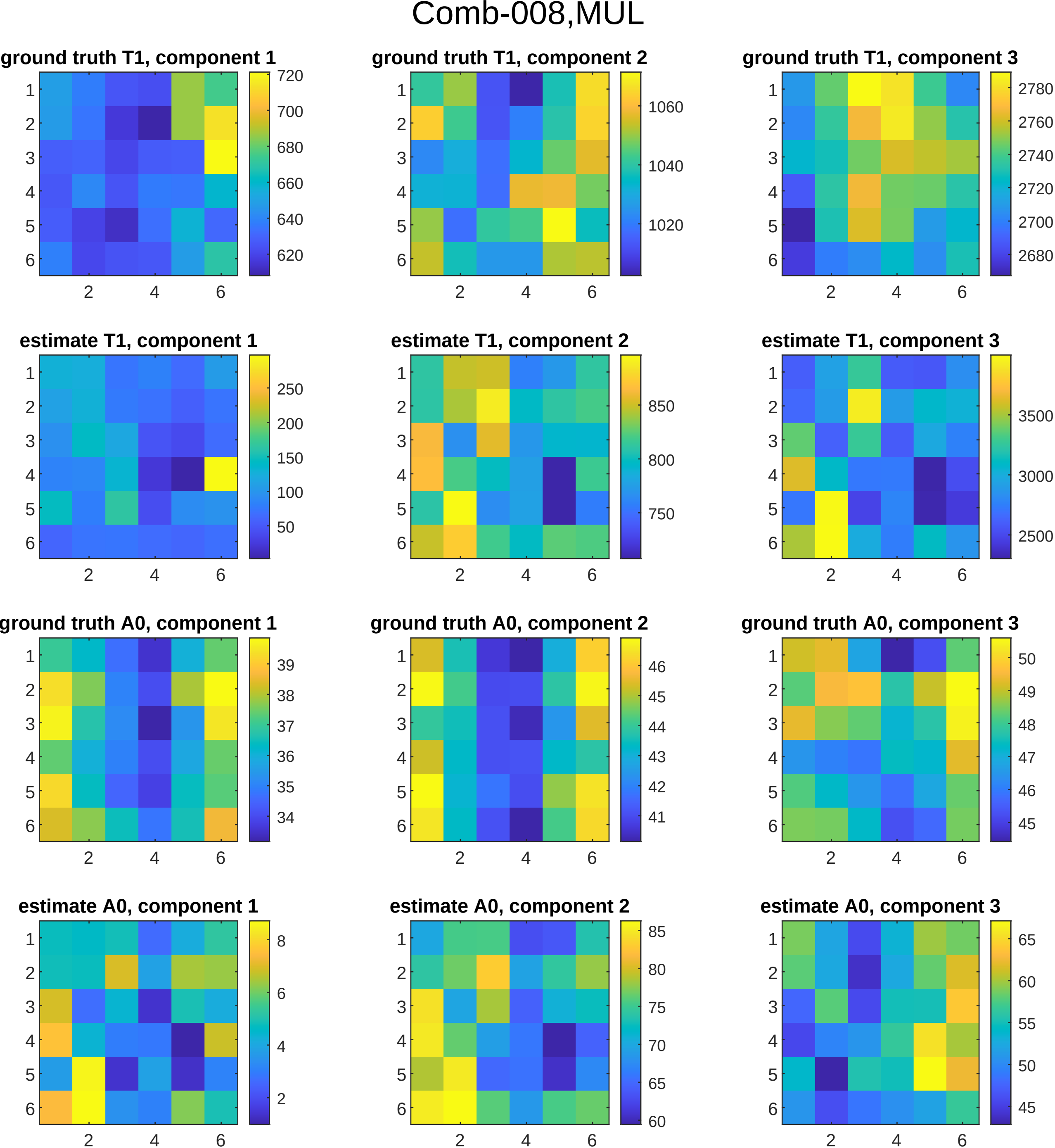
Comb-008,MUL
ground truth T1, component 1
720
700
680
660
640
620
ground truth T1, component 2
1060
1040
1020
ground truth T1, component 3
2780
2760
2740
2720
2700
2680
estimate T1, component 1
250
200
150
100
50
estimate T1, component 2
850
800
750
estimate T1, component 3
3500
3000
2500
ground truth A0, component 1
39
38
37
36
35
34
ground truth A0, component 2
46
45
44
43
42
41
ground truth A0, component 3
50
49
48
47
46
45
estimate A0, component 1
8
6
4
2
estimate A0, component 2
85
80
75
70
65
60
estimate A0, component 3
65
60
55
50
45

Comb-008,TOM

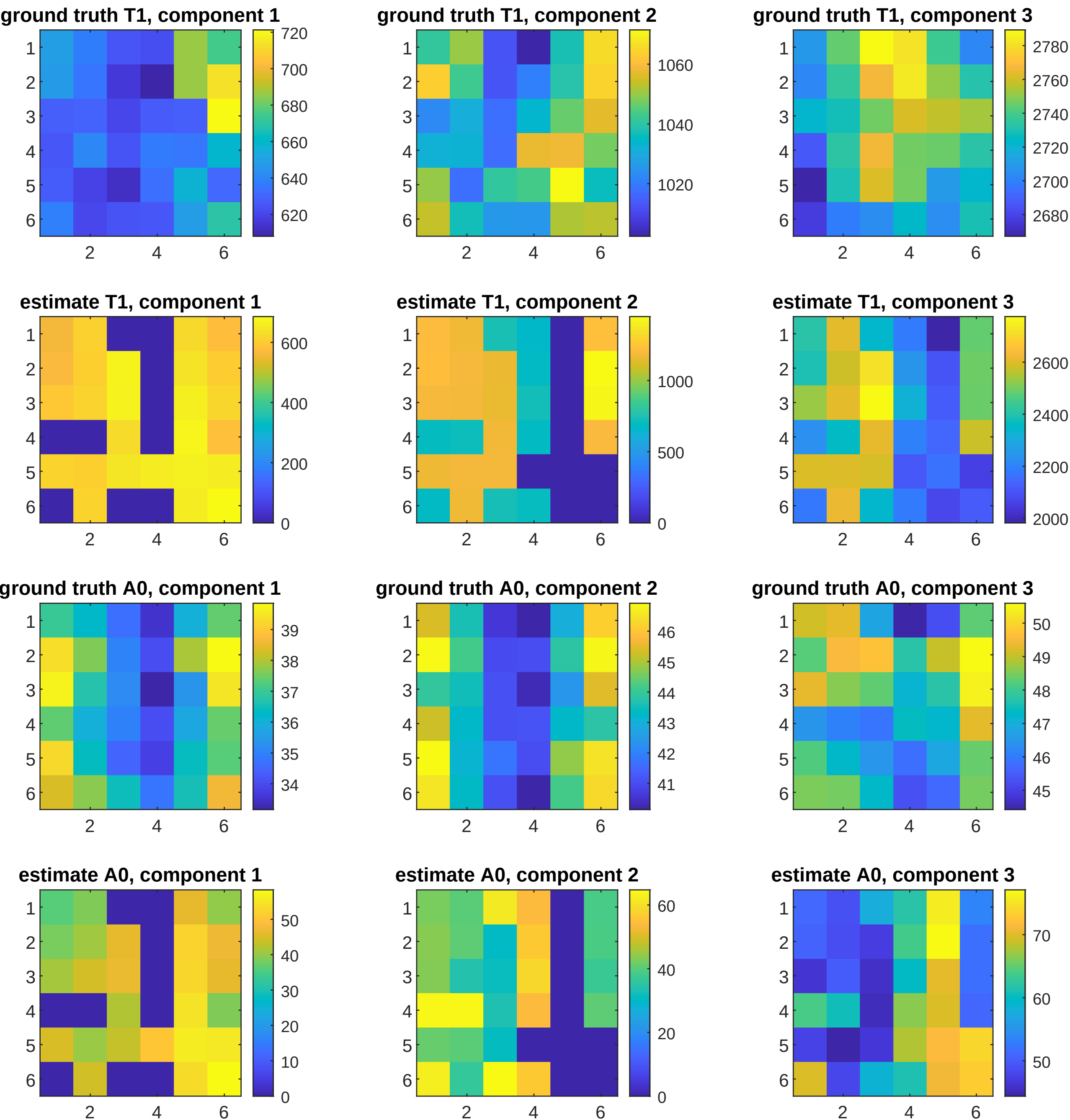
ground truth T1, component 1
ground truth T1, component 2
ground truth T1, component 3
estimate T1, component 1
estimate T1, component 2
estimate T1, component 3
ground truth A0, component 1
ground truth A0, component 2
ground truth A0, component 3
estimate A0, component 1
estimate A0, component 2
estimate A0, component 3

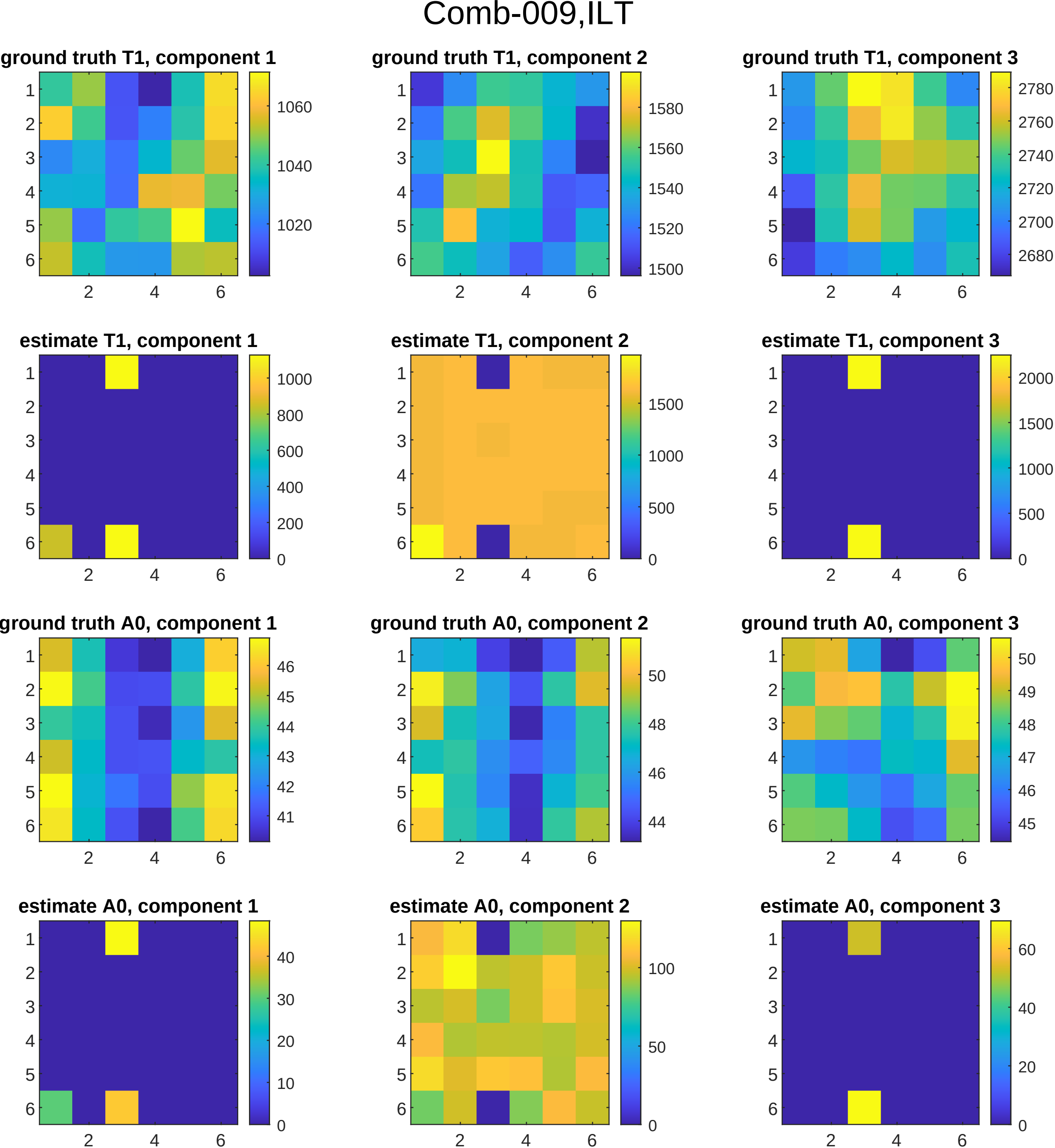
Comb-009,ILT
ground truth T1, component 1
ground truth T1, component 2
ground truth T1, component 3
estimate T1, component 1
estimate T1, component 2
estimate T1, component 3
ground truth A0, component 1
ground truth A0, component 2
ground truth A0, component 3
estimate A0, component 1
estimate A0, component 2
estimate A0, component 3

Comb-009,MUL

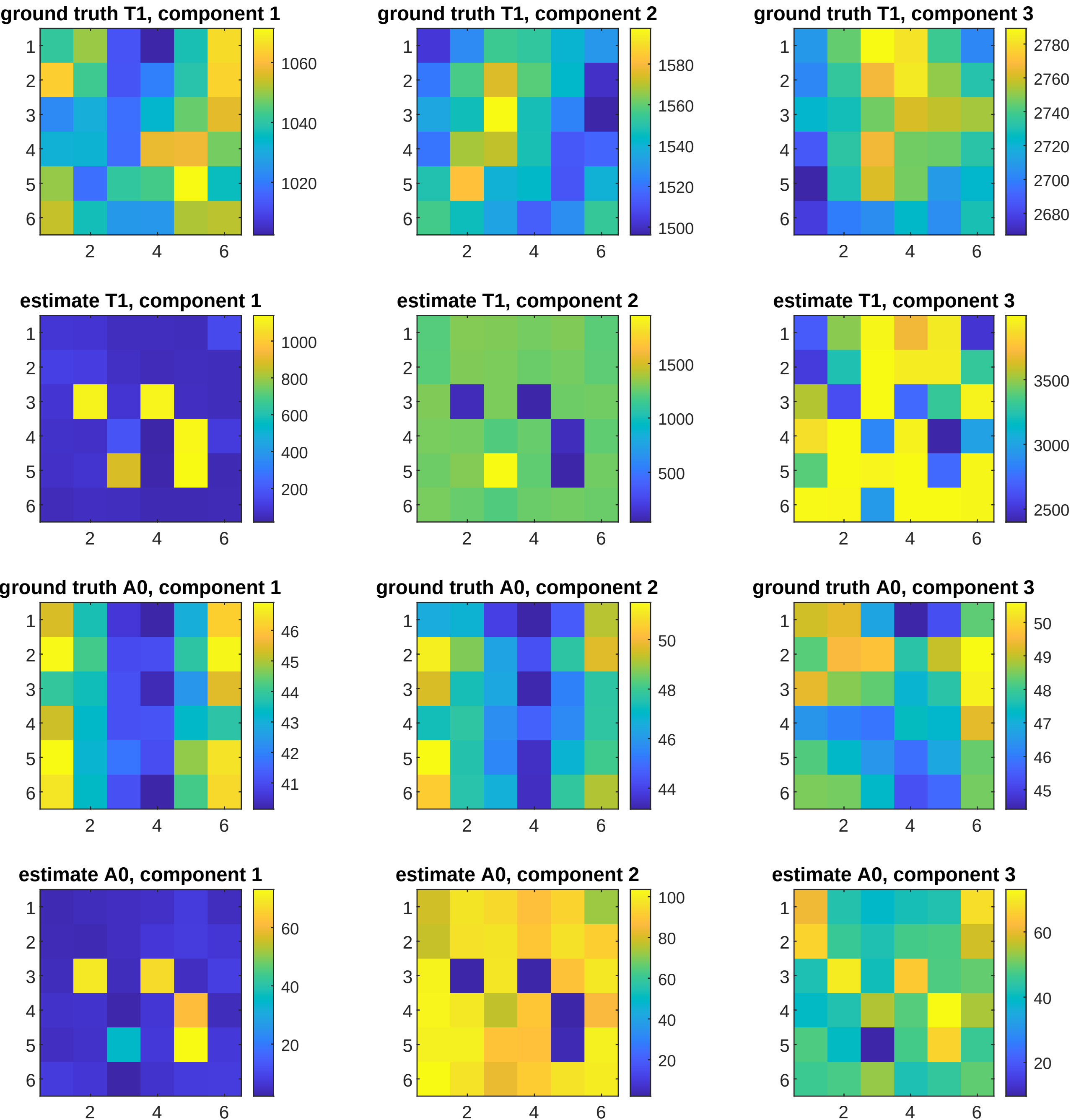
ground truth T1, component 1
1060
1040
1020
ground truth T1, component 2
1580
1560
1540
1520
1500
ground truth T1, component 3
2780
2760
2740
2720
2700
2680
estimate T1, component 1
1000
800
600
400
200
estimate T1, component 2
1500
1000
500
estimate T1, component 3
3500
3000
2500
ground truth A0, component 1
46
45
44
43
42
41
ground truth A0, component 2
50
48
46
44
ground truth A0, component 3
50
49
48
47
46
45
estimate A0, component 1
60
40
20
estimate A0, component 2
100
80
60
40
20
estimate A0, component 3
60
40
20

Comb-009,TOM

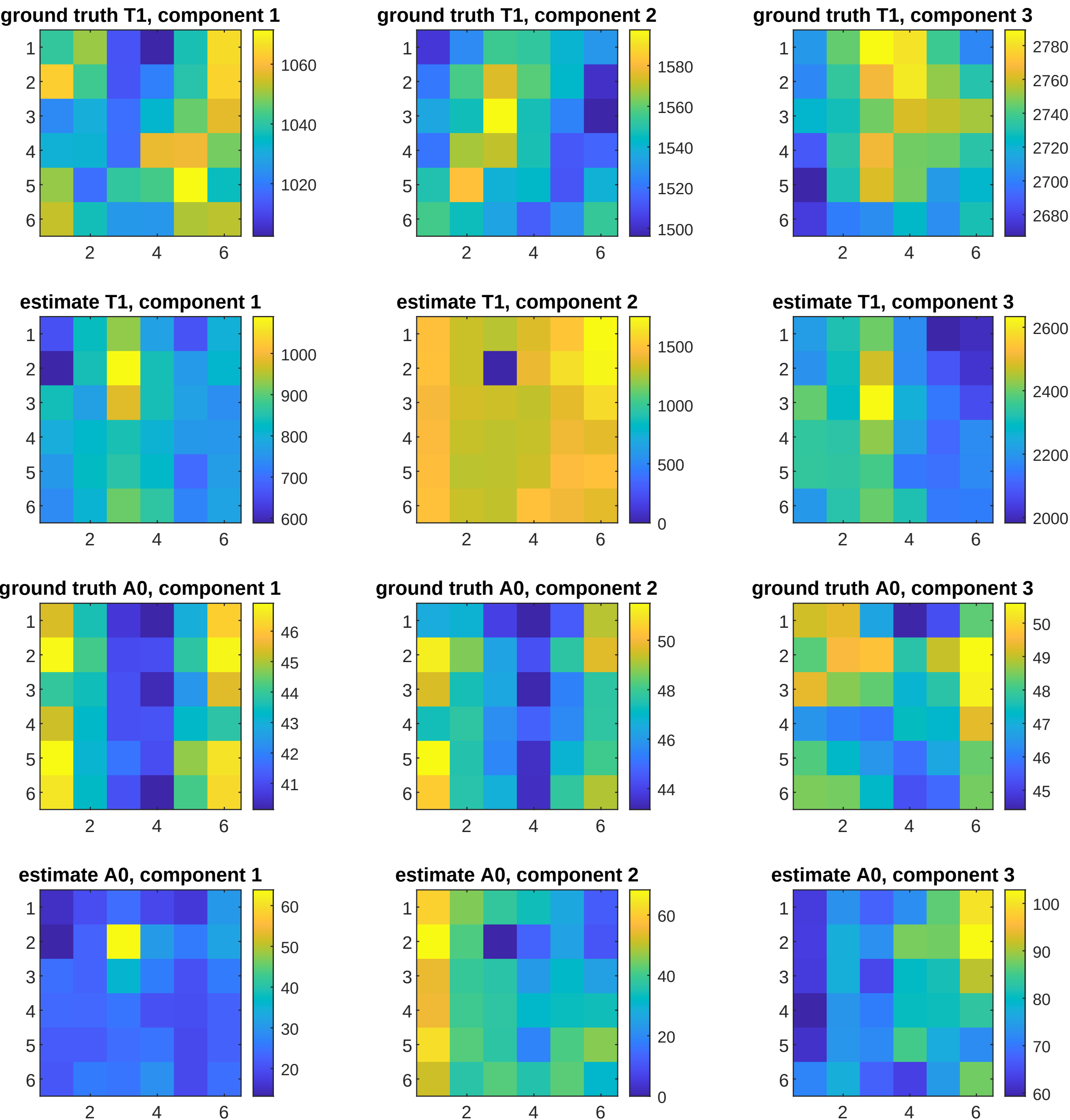
ground truth T1, component 1
1060
1040
1020
ground truth T1, component 2
1580
1560
1540
1520
1500
ground truth T1, component 3
2780
2760
2740
2720
2700
2680
estimate T1, component 1
1000
900
800
700
600
estimate T1, component 2
1500
1000
500
0
estimate T1, component 3
2600
2400
2200
2000
ground truth A0, component 1
46
45
44
43
42
41
ground truth A0, component 2
50
48
46
44
ground truth A0, component 3
50
49
48
47
46
45
estimate A0, component 1
60
50
40
30
20
estimate A0, component 2
60
40
20
0
estimate A0, component 3
100
90
80
70
60
1
2
3
4
5
6
2
4
6

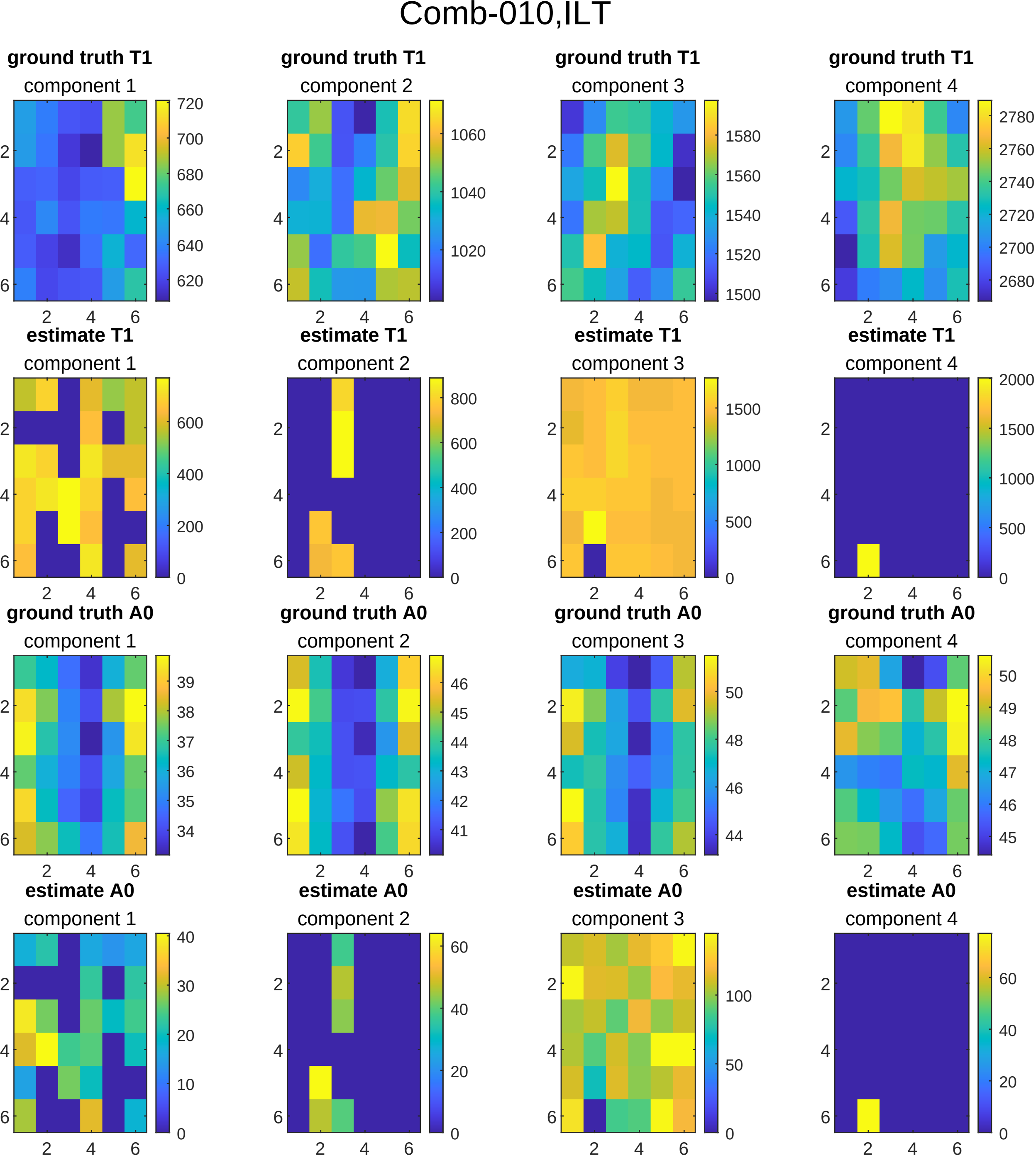

Comb-010,ILT
ground truth T1
component 1
component 2
component 3
component 4
estimate T1
ground truth A0
estimate A0

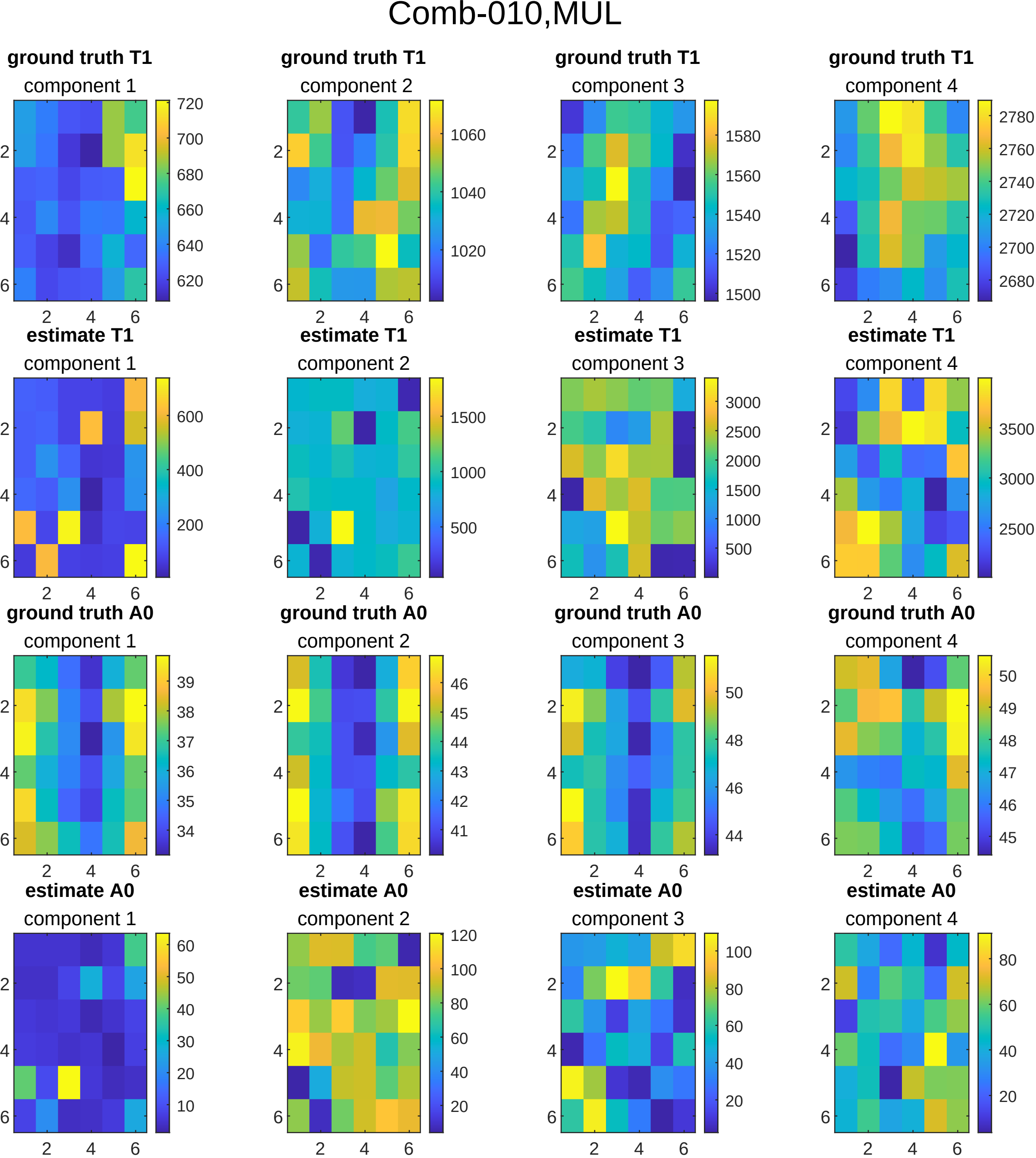
Comb-010,MUL
ground truth T1
component 1
ground truth T1
component 2
ground truth T1
component 3
ground truth T1
component 4
estimate T1
component 1
estimate T1
component 2
estimate T1
component 3
estimate T1
component 4
ground truth A0
component 1
ground truth A0
component 2
ground truth A0
component 3
ground truth A0
component 4
estimate A0
component 1
estimate A0
component 2
estimate A0
component 3
estimate A0
component 4

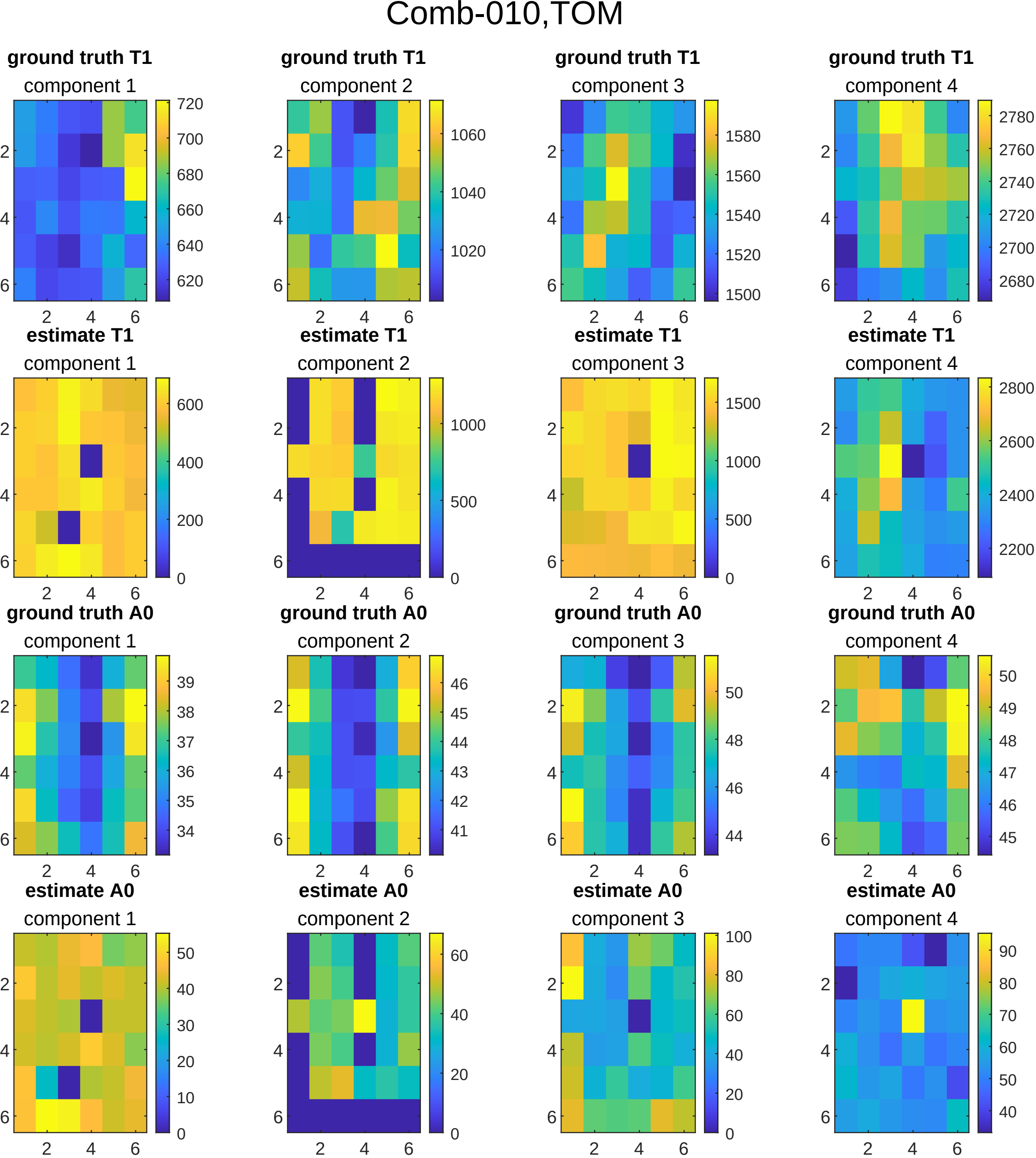
Comb-010,TOM
ground truth T1
component 1
component 2
component 3
component 4
estimate T1
ground truth A0
estimate A0
