# Supplementary material for: Limitations of multiexponential T1 mapping of cortical myeloarchitecture
Source: PLoS One. 2025 Dec 4;20(12):e0338035. doi: 10.1371/journal.pone.0338035 (PMC12677506; doi:10.1371/journal.pone.0338035)
Supplement: S6 File — (PDF) [file pone.0338035.s006.pdf]

Mean absolute values ± standard deviation of ground truth and estimates for all composite data sets.

**Dataset ID**	**N of components**	**Component ID**	**Method**	**T1 ground truth [ms]**	**T1 estimate [ms]**	**A0 ground truth [-]**	**A0 estimate [-]**
1	2	1	ILT	641 ± 27	66 ± 168	36 ± 2	2 ± 6
1	2	2	ILT	1039 ± 17	878 ± 58	43 ± 2	59 ± 7
1	2	1	MUL	641 ± 27	329 ± 131	36 ± 2	10 ± 8
1	2	2	MUL	1039 ± 17	918 ± 73	43 ± 2	70 ± 6
1	2	1	TOM	641 ± 27	795 ± 29	36 ± 2	72 ± 6
1	2	2	TOM	1039 ± 17	1517 ± 239	43 ± 2	7 ± 5
2	2	1	ILT	641 ± 27	373 ± 178	36 ± 2	10 ± 6
2	2	2	ILT	1540 ± 23	1182 ± 64	47 ± 2	57 ± 8
2	2	1	MUL	641 ± 27	568 ± 63	36 ± 2	29 ± 6
2	2	2	MUL	1540 ± 23	1441 ± 86	47 ± 2	54 ± 7
2	2	1	TOM	641 ± 27	1031 ± 9	36 ± 2	77 ± 4
2	2	2	TOM	1540 ± 23	2131 ± 74	47 ± 2	3 ± 1
3	2	1	ILT	641 ± 27	607 ± 39	36 ± 2	21 ± 4
3	2	2	ILT	2733 ± 29	2169 ± 181	48 ± 2	44 ± 7
3	2	1	MUL	641 ± 27	569 ± 23	36 ± 2	32 ± 2
3	2	2	MUL	2733 ± 29	2556 ± 84	48 ± 2	52 ± 3
3	2	1	TOM	641 ± 27	624 ± 153	36 ± 2	34 ± 6
3	2	2	TOM	2733 ± 29	2565 ± 75	48 ± 2	49 ± 8
4	2	1	ILT	1039 ± 17	0 ± 0	43 ± 2	0 ± 0
4	2	2	ILT	1540 ± 23	1272 ± 24	47 ± 2	70 ± 9
4	2	1	MUL	1039 ± 17	607 ± 368	43 ± 2	20 ± 28
4	2	2	MUL	1540 ± 23	1540 ± 507	47 ± 2	70 ± 29
4	2	1	TOM	1039 ± 17	1196 ± 28	43 ± 2	78 ± 5
4	2	2	TOM	1540 ± 23	1962 ± 173	47 ± 2	12 ± 3
5	2	1	ILT	1039 ± 17	833 ± 549	43 ± 2	18 ± 16
5	2	2	ILT	2733 ± 29	1988 ± 280	48 ± 2	53 ± 16
5	2	1	MUL	1039 ± 17	839 ± 66	43 ± 2	28 ± 5
5	2	2	MUL	2733 ± 29	2351 ± 130	48 ± 2	62 ± 6

Dataset ID	N of components	Component ID	Method	T1 ground truth [ms]	T1 estimate [ms]	A0 ground truth [-]	A0 estimate [-]
5	2	1	TOM	1039 ± 17	872 ± 89	43 ± 2	30 ± 5
5	2	2	TOM	2733 ± 29	2373 ± 123	48 ± 2	61 ± 7
6	2	1	ILT	1540 ± 23	280 ± 706	47 ± 2	11 ± 28
6	2	2	ILT	2733 ± 29	1763 ± 719	48 ± 2	64 ± 28
6	2	1	MUL	1540 ± 23	692 ± 224	47 ± 2	8 ± 4
6	2	2	MUL	2733 ± 29	2228 ± 86	48 ± 2	87 ± 5
6	2	1	TOM	1540 ± 23	1474 ± 89	47 ± 2	30 ± 3
6	2	2	TOM	2733 ± 29	2416 ± 127	48 ± 2	63 ± 4
7	3	1	ILT	641 ± 27	104 ± 202	36 ± 2	5 ± 10
7	3	2	ILT	1039 ± 17	879 ± 439	43 ± 2	78 ± 40
7	3	3	ILT	1540 ± 23	237 ± 490	47 ± 2	16 ± 34
7	3	1	MUL	641 ± 27	207 ± 269	36 ± 2	13 ± 19
7	3	2	MUL	1039 ± 17	692 ± 366	43 ± 2	61 ± 35
7	3	3	MUL	1540 ± 23	1782 ± 534	47 ± 2	54 ± 22
7	3	1	TOM	641 ± 27	580 ± 47	36 ± 2	33 ± 4
7	3	2	TOM	1039 ± 17	1220 ± 34	43 ± 2	84 ± 9
7	3	3	TOM	1540 ± 23	1975 ± 176	47 ± 2	10 ± 5
8	3	1	ILT	641 ± 27	616 ± 226	36 ± 2	28 ± 12
8	3	2	ILT	1039 ± 17	84 ± 242	43 ± 2	5 ± 15
8	3	3	ILT	2733 ± 29	1690 ± 138	48 ± 2	67 ± 11
8	3	1	MUL	641 ± 27	90 ± 50	36 ± 2	5 ± 2
8	3	2	MUL	1039 ± 17	810 ± 44	43 ± 2	73 ± 7
8	3	3	MUL	2733 ± 29	2939 ± 443	48 ± 2	54 ± 6
8	3	1	TOM	641 ± 27	454 ± 287	36 ± 2	33 ± 22
8	3	2	TOM	1039 ± 17	771 ± 504	43 ± 2	35 ± 23
8	3	3	TOM	2733 ± 29	2365 ± 220	48 ± 2	58 ± 10
9	3	1	ILT	1039 ± 17	86 ± 292	43 ± 2	3 ± 12
9	3	2	ILT	1540 ± 23	1550 ± 386	47 ± 2	96 ± 26
9	3	3	ILT	2733 ± 29	125 ± 521	48 ± 2	3 ± 14
9	3	1	MUL	1039 ± 17	210 ± 357	43 ± 2	13 ± 20

Dataset ID	N of components	Component ID	Method	T1 ground truth [ms]	T1 estimate [ms]	A0 ground truth [-]	A0 estimate [-]
9	3	2	MUL	1540 ± 23	1156 ± 406	47 ± 2	83 ± 30
9	3	3	MUL	2733 ± 29	3510 ± 566	48 ± 2	49 ± 12
9	3	1	TOM	1039 ± 17	803 ± 90	43 ± 2	25 ± 8
9	3	2	TOM	1540 ± 23	1371 ± 264	47 ± 2	36 ± 15
9	3	3	TOM	2733 ± 29	2246 ± 143	48 ± 2	76 ± 10
10	4	1	ILT	641 ± 27	421 ± 325	36 ± 2	15 ± 13
10	4	2	ILT	1039 ± 17	135 ± 306	43 ± 2	8 ± 18
10	4	3	ILT	1540 ± 23	1463 ± 259	47 ± 2	109 ± 26
10	4	4	ILT	2733 ± 29	56 ± 334	48 ± 2	2 ± 13
10	4	1	MUL	641 ± 27	212 ± 224	36 ± 2	11 ± 13
10	4	2	MUL	1039 ± 17	828 ± 331	43 ± 2	78 ± 32
10	4	3	MUL	1540 ± 23	1835 ± 908	47 ± 2	46 ± 32
10	4	4	MUL	2733 ± 29	3033 ± 616	48 ± 2	46 ± 19
10	4	1	TOM	641 ± 27	574 ± 146	36 ± 2	40 ± 11
10	4	2	TOM	1039 ± 17	740 ± 577	43 ± 2	26 ± 21
10	4	3	TOM	1540 ± 23	1478 ± 279	47 ± 2	54 ± 19
10	4	4	TOM	2733 ± 29	2420 ± 149	48 ± 2	53 ± 10
