# Supplementary material for: Limitations of multiexponential T1 mapping of cortical myeloarchitecture
Source: PLoS One. 2025 Dec 4;20(12):e0338035. doi: 10.1371/journal.pone.0338035 (PMC12677506; doi:10.1371/journal.pone.0338035)
Supplement: Attachment — Submitted filename: response_to_reviewers_revision-01.docx [file pone.0338035.s007.docx]

Limitations of multiexponential *T_1_* mapping of cortical myeloarchitecture

# Response to Editors’ comments

**Point 1:** PLOS ONE's style requirements

**Response 1:** We have revised the manuscript, figures, and tables and updated all documents according to PLOS ONE's style requirements.

**Point 2:** Financial disclosure

**Response 2:** We have included the amended Role of Funder statement, stating: "The funders had no role in study design, data collection and analysis, decision to publish, or preparation of the manuscript.", in our cover letter. We have also disclosed additional funding information for two authors, who did not provide this information during the initial submission. As this is not possible to amend in the submission system, we have included the complete up to date funding information in the cover letter.

**Point 3:** Acceptable data repository

**Response 3:** We have changed the data repository to Zenodo as per PLOS ONE recommended repositories.

**Point 4:** Data availability statement

**Response 4:** We have uploaded the data to Zenodo and locked the data access pending acceptance of the manuscript for publication. The data can be accessed from https://doi.org/10.5281/zenodo.17456443.

**Point 5:** Recommendations to cite specific literature

**Response 5:** No recommendations to cite specific literature were made by the reviewers.

**Point 6:** Reference list

**Response 6:** We have reviewed the reference list ensuring that it is complete and up to date. No references were retracted.

# Response to Reviewer 1 comments

Dear reviewer, thank you for your valuable comments and insights, which helped us improve our manuscript.

**Point 1:** Dear authors, please provide a more concrete conclusion, which should also emphasize the clinical relevance. The lowest relative error achieved across multiexponential models was around 20%, what expectations were at the beginning of the study? If visualization of cortical layers based on these longitudinal relaxation-time estimates is unlikely to represent their true structure, then what clinical importance does this input in future research?

**Response 1:** At the outset of our experiments, we anticipated that the achievable precision of multiexponential decomposition would be limited by the ill-posed nature of the inverse problem and by the relatively narrow range of T₁ values encountered in the brain cortex. The lowest relative error of approximately 20% observed in our results was consistent with our expectations and with the theoretical limits of the method. However, as the modeling represents only the first step in a longer processing pipeline, additional errors are inevitably introduced in subsequent delineation and visualization steps. Therefore, the modeling itself would need to achieve substantially lower error—on the order of 5%—to ensure clinically meaningful precision, underscoring why the multiexponential approach at 3 T is unlikely to yield anatomically valid layer maps.

Clinical importance of our results is twofold. First, as our findings give estimates on the expected precision which can be achieved via multiexponential modeling, they will help clinical researchers better assess the credibility of any published research based on this methodological framework and evaluate whether to employ it in their own research. Second, as the estimation accuracy decreases with the number of expected cortical layers our findings suggest that the multiexponential approach is more suitable for examination of allocortex as opposed to the neocortex. Whether this could form a basis for successful clinical applications, for example for the early detection of Alzheimer’s disease via *in vivo* examination of entorhinal regions, remains to be seen in future research.

We have updated the conclusion to better reflect the above-mentioned and as such to deliver a more concrete message (line 429-436).

# Response to Reviewer 2 comments

The manuscript is well written with enough details for reader to understand and reproduce. The hypothesis is clearly presented, and the data supports the conclusions.

Dear reviewer, thank you for reading our manuscript and providing us with your positive and encouraging feedback. We believe that after the changes made to address yours and Reviewer 1`s comments, the manuscript is now even better.

**Point 1:** A major concern I have is the data were added in the image domain to generate composite signals. In MRI the data (composite signal) is acquired in frequency domain and then transformed to image domain for fitting. I do not expect that this will change the conclusions, but it should be addressed in the discussions.

**Response 1:** We have addressed the issue of addition in the composite domain as opposed to frequency domain in the discussion (line 388-391). As we have employed Cartesian sampling the relationship between frequency and image domains is given by the Fourier transform which is a linear operator thus preserving the operation of addition. We therefore believe that our simplification does not significantly hamper generalizability of our experiment.

**Point 2:** The results are presented in terms of relative error. It would also be useful to show a table listing ground truth T1 and estimated T1 and standard deviation or error based on 2, 3, and 4 component composite data.

**Response 2:** We have added a table with mean and standard deviation for ground truth values and parameter estimates, for all composite data sets, to the supplementary materials, as this table is rather large (see supplementary material S6, referenced in manuscript text on line 272-274).

**Point 3:** Equation (2) is valid for 180-degree inversion. Otherwise, there will be additional fitting variable. Was inversion efficiency verified?

**Response 3:** The inversion pulse was automatically set and verified by the scanner, with the phantom placed in the isocenter to further ensure maximal inversion efficiency. We have added the preceding statement to the methods section of the manuscript (line 148-149) to make this information clear to the reader.

**Point 4:** Line 136 and Table 1: Seven different MNCl2 concentrations were prepared but why table 2 only uses five different T1s to generate composite signal.

**Response 4:** This is due to the low signal quality and therefore unreliable ground-truth T_1_ estimate of the remaining ROI (this fact is mentioned in the methods sections titled *Ground truth* – line 160-161) which prevented us from making additional meaningful ROI combinations. We have added clarification to Table 1. caption to make this fact more easily available to the reader (line 174-175).

**Point 5:** It would be helpful to show the parameter maps, and ROI.

**Response 5:** We have added ground truth and parametric estimate maps for the whole image and the individual ROI to the supplementary materials (see supplementary material S5, referenced in manuscript text on line 272-274). As each ground truth constitutes a 6 x 6 square extracted from a mostly homogeneous area and the parameter estimates can vary substantially, each image must be interpreted w. r. t. to its individual pseudocolor scale.
